# The role of anthropogenic dispersal in shaping the distribution and genetic composition of a widespread North American tree species

**DOI:** 10.1002/ece3.7944

**Published:** 2021-07-30

**Authors:** Graham E. Wyatt, J. L. Hamrick, Dorset W. Trapnell

**Affiliations:** ^1^ Department of Plant Biology University of Georgia Athens Georgia USA

**Keywords:** *Asimina triloba*, clonality, genetic structure, pawpaw, population genetics, pre‐Columbian peoples

## Abstract

Dispersal and colonization are among the most important ecological processes for species persistence as they allow species to track changing environmental conditions. During the last glacial maximum (LGM), many cold‐intolerant Northern Hemisphere plants retreated to southern glacial refugia. During subsequent warming periods, these species expanded their ranges northward. Interestingly, some tree species with limited seed dispersal migrated considerable distances after the LGM ~19,000 years before present (YBP). It has been hypothesized that indigenous peoples may have dispersed valued species, in some cases beyond the southern limits of the Laurentide Ice Sheet. To investigate this question, we employed a molecular genetics approach on a widespread North American understory tree species whose fruit was valued by indigenous peoples. Twenty putative anthropogenic (near pre‐Columbian habitations) and 62 wild populations of *Asimina triloba* (pawpaw), which produces the largest edible fruit of any North American tree, were genetically assayed with nine microsatellite loci. Putative anthropogenic populations were characterized by reduced genetic diversity and greater excess heterozygosity relative to wild populations. Anthropogenic populations in regions that were glaciated during the LGM had profiles consistent with founder effects and reduced gene flow, and shared rare alleles with wild populations hundreds of kilometers away (mean = 723 km). Some of the most compelling evidence for human‐mediated dispersal is that putative anthropogenic and wild populations sharing rare alleles were separated by significantly greater distances (mean = 695 km) than wild populations sharing rare alleles (mean = 607 km; *p* = .014). Collectively, the genetic data suggest that long‐distance dispersal played an important role in the distribution of pawpaw and is consistent with the hypothesized role of indigenous peoples.

## Graphical abstract

We employ molecular genetics to investigate hypothesized anthropogenic dispersal of *Asimina triloba* (pawpaw), a valued North American tree species, by pre‐Columbian peoples. Levels and partitioning of genetic variation in 20 putative anthropogenic and 62 wild populations reveal intriguing patterns, namely that putative anthropogenic and wild populations have distinctly different genetic signatures that are consistent with, and may provide clues to, anthropogenic dispersal.

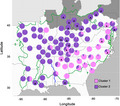

## 1 INTRODUCTION

Dispersal and colonization are critical ecological processes that affect the ability of organisms to undergo range shifts as they track a changing environment and thus to persist over evolutionary time scales. During ice age glacier advances, most cold‐intolerant Northern Hemisphere plant species retreated to southern refugia and during subsequent warming periods there was extensive northward migration and often range expansion (Davis & Shaw, 2001; Hewitt, 2000; Lyford et al., 2003). Understanding historical patterns of plant dispersal and mechanisms responsible for movement during the warming climate that ensued after the last glacial maximum (LGM; Clark et al., 2009) allows inferences and predictions regarding their capacity for range modification in response to future climatic shifts.

Sessile plants disperse their seeds by abiotic means (i.e., barochory, hydrochory, or anemochory) and/or biotic vectors (epi‐ and endozoochory) with some species having more than one dispersal mechanism. Interestingly, some taxa with limited seed dispersal, such as large‐seeded tree species without an obvious dispersal vector, appear to have migrated considerable distances after glacial retreat in the late Pleistocene (~19,000 years before present [YBP]), a phenomenon known as “Reid's Paradox” (Clark et al., 1998; MacDougall, 2003). In 1899, Clement Reid was the first of many to note the surprisingly rapid rate of northward migration from glacial refugia by large‐seeded trees, such as oaks, into formerly glaciated regions of Great Britain (Clark et al., 1998). Some have hypothesized that humans may have mediated dispersal of valued tree species beyond the limits of their southern glacial refugia (Abrams & Nowacki, 2008; Delcourt & Delcourt, 2004; MacDougall, 2003).

There is considerable evidence that pre‐Columbian peoples had a marked impact on the North American landscape prior to European arrival (Abrams & Nowacki, 2008; Denevan, 1992; Pyne, 1983; Stewart, 2002). However, little attention has been paid to the possible role of native peoples in the dispersal of useful plant species (but see Abrams & Nowacki, 2008; Cottrell et al., 2002; Delcourt & Delcourt, 2004; MacDougall, 2003; Petit et al., 2002) and some population genetic studies have intentionally avoided species valued or impacted by humans (Petit et al., 2003). One of the few exceptions involving a North American tree species used genetic markers and packrat middens (pollen and macrofossils) to investigate an isolated (>200 km) northern population of *Pinus edulis* (Colorado pinyon pine: Bentancourt et al., 1991). The authors concluded that this isolated population originated by long‐distance dispersal by Native Americans who valued pine nuts as a food source. Additional phytogeographic evidence of human‐mediated dispersal in North America comes from tobacco (*Nicotiana* sp.) and bottle gourd (*Lagenaria siceraria*; Asch, 1995). Both species are thought to have been absent from the area presently known as Illinois (U.S.A.) until their introduction by external contacts and subsequent cultivation as useful specialty‐plant species. Wykoff (1991) asserted that black walnut (*Juglans nigra*), some hickories (*Carya* spp.), oaks (*Quercus* spp.), and medicinal plant species were likely introduced into what is now recognized as New York state (U.S.A.) prior to European contact.

At least 15,000 YBP an ice‐free corridor connected Alaska and the rest of the continental United States, permitting human migration into this region. Earlier arrival of humans in North America ~16,000 YBP could also have been possible along the North Pacific coast (Erlandson et al., 2015). It is generally agreed that indigenous peoples inhabiting North America manipulated their environment to manage resources soon after their arrival ~15,000 (Delcourt & Delcourt, 2004; Morse & Morse, 1983) to 17,000 YBP (Adovasio et al., 1978; Richland et al., 2007); however, debate remains about the uniformity of their influence over space and time (Muñoz et al., 2014).

While Denevan (2011) estimated two million indigenous people lived in North America in 1492, when Europeans commenced colonization, there is much controversy over the extent of anthropogenic impact on the present distribution of plant species. Some investigators maintain that impacts by indigenous peoples were ubiquitous, with few ecosystems unaffected (Abrams & Nowacki, 2008; Denevan, 1992; Kay, 2002; Krech, 1999). However, after the introduction of Old World diseases by Spanish explorers and the resulting decimation of indigenous populations (Lovell, 1992), North American ecosystems recovered somewhat, which may have obscured indigenous imprints on the landscape (Denevan, 1992). Others have argued that landscape alteration near densely settled areas showed greater impacts, while more remote, sparsely inhabited areas exhibited little anthropogenic alteration (Muñoz et al., 2014; Parker, 2002; Vale, 1998, 2002). Thus, plants used by indigenous people may show signs of manipulation in proximity to settlements but may exist in an un‐manipulated state farther away (e.g., Parker et al., 2014). Muñoz et al. (2014) contended that agricultural and silvicultural impacts were greatest near settlements, as well as along riparian corridors and along trade routes. By approximately 2,000 YBP, indigenous societies throughout the river valleys of Eastern North America were interacting widely, erecting monuments, and producing sophisticated material cultures (Wright, 2017). Various land‐use patterns exhibited by indigenous peoples include the following: (a) semi‐nomadic hunter‐gatherers with temporary encampments, (b) seasonal movements and periodic occupation of resource‐specific sites with hunting grounds, nut trees, fish, and/or shellfish, and (c) stable villages associated with agricultural fields or aquatic food resources (Abrams & Nowacki, 2008). Highlands and Piedmont Lowlands were characterized by temporary seasonal encampments (Pagoulatos, 1992). More stable villages were located along major rivers and tributaries of Ridge and Valley areas and the Coastal Plain, while foraging destinations were scattered across the landscape. Hunter‐gatherer groups were more typical in the north, while more agricultural‐based subsistence was practiced from Massachusetts southward, especially along major river valleys (Patterson & Sassaman, 1988). By 2,000 YBP, large agricultural fields associated with settlements were established in the floodplains of larger rivers in the Midwest and the south (Fritz, 1990; Scarry & Scarry, 2005). Some cultures relied heavily on nutritious nuts (i.e., balanocultures) of mast‐producing species such as chestnut, hickory, and oaks and are thought to have planted orchards and manipulated the distribution of some tree species (Abrams & Nowacki, 2008). Acorns served as a particularly important dietary staple for many indigenous peoples (Bainbridge, 1985; Logan, 2005), and widespread acorn use almost certainly predates widespread corn use (Bainbridge, 1985). There were many other crops that were cultivated as well (e.g., Delcourt & Delcourt, 2004; Mueller, 2017; Schroeder, 1999). Inadvertent or intentional anthropogenic seed dispersal, particularly in heavily impacted portions of the landscape, may have contributed to postglacial maximum range expansion (e.g., Abrams & Nowacki, 2008; Keener & Kuhns, 1998; White, 1906; Wykoff, 1991) and help explain Reid's Paradox. It is important that this question be investigated in more depth in order to develop a better understanding of plant dispersal and factors that have shaped the distribution of plant taxa.

*Asimina triloba* (Annonaceae), whose common name is pawpaw or Indian banana, has the largest (5–16 cm long, 3–7 cm wide) edible fruits of any native North American tree. The flavorful fruits (described as a cross between banana, mango, and pineapple) were prized as a food source by native peoples (e.g., Brooks & Johannes, 1990; Keener & Kuhns, 1998; Peterson, 1991), and the fibrous bark was used to make rope and cloth (Peattie, 1950). It may also have been utilized medicinally (Krochmal & Krochmal, 1973; Peterson, 1991). In recent years, there has been considerable research on promising anti‐cancer properties of *A. triloba* seeds and bark (e.g., Zhao et al., 1993).

Seeds or carbonized remains of *A*. *triloba* have been found along the Mississippi and Ohio Rivers and at numerous archaeological sites in Arkansas, Illinois, Indiana, Kansas, Kentucky, Mississippi, and Missouri (Blasing, 1986; Cutler & Blake, 1976; Gilmore, 1931; Jones, 1936; King, 1982; Waselkov, 1977; Wedel, 1943; Yarnell & Watson, 1966) and may have been dispersed by various cultures (Brooks & Johannes, 1990; Keener & Kuhns, 1998; Peterson, 1991; Wykoff, 1991). The first written record of *A*. *triloba* comes from Hernando de Soto's expedition in 1541 across Southeastern North America, which reported widespread planting of the tree by indigenous tribes of the southeast and mentions its flavor and fragrance (Pickering, 1879). Some authors have asserted that *A*. *triloba* was grown by pre‐Columbian people (Cai et al., 2019; Hormaza, 2004; Peattie, 1950; Peterson, 1991), although there is no known evidence for the cultivation of *A*. *triloba* orchards by Native Americans beyond such written reports. There is however molecular evidence for pre‐Columbian dispersal of *Annona cherimola* between Central and South America (Larranaga et al., 2017), a species that shares some attributes of *A. triloba* (i.e., a woody perennial that produces large, edible, and nutritious fruits).

It has been hypothesized that the remarkably wide distribution of pawpaw throughout much of eastern North America relative to its seven North American congeners, which are restricted to the southeastern United States, is due in part to its spread by indigenous peoples who may have actively traded propagules, encouraged its growth (e.g., Abrams & Nowacki, 2008; Keener & Kuhns, 1998; MacDougall, 2003), and/or inadvertently dispersed its seeds (White, 1906). White (1906) for example observed Native Americans using pawpaw seeds as game pieces, contending that this game is centuries old and that Sac and Fox Indians may have unintentionally dispersed the seeds. That the fruit cannot easily be stored or transported suggests the advantages of planting pawpaw seeds along well‐traveled routes and/or near settlements. Its occurrence north of the southern Pleistocene glacial boundary suggests that its post‐LGM dispersal could have been human‐mediated. While pawpaw was clearly used by indigenous peoples in North America, there has been debate regarding the relative importance of natural versus anthropogenic dispersal (Keener & Kuhns, 1998; Murphy, 2001) with more authors favoring an anthropogenic role (e.g., Abrams & Nowacki, 2008; Keener & Kuhns, 1998; MacDougall, 2003).

The overarching goal of our research is to employ a molecular genetics approach to test the hypothesis that indigenous pre‐Columbian people contributed to the dispersal and range expansion of a valued North American understory tree. To address this question, we used genetic signatures contained within contemporary *A*. *triloba* populations of wild and putative anthropogenic origin to assess levels of genetic diversity and test whether patterns of genetic variation within and among populations across the species’ range are consistent with anthropogenic dispersal. Specifically, we (a) estimate and compare levels of genetic diversity of wild populations and those of putative anthropogenic origin (i.e., populations occurring near known Native American settlements), and (b) test whether patterns of genetic structure among populations are consistent with the hypothesis of anthropogenic dispersal, particularly into regions glaciated during the LGM. Our a priori expectation is that if humans dispersed *A*. *triloba* that resulting populations would be characterized by lower genetic diversity because of founder effects and higher genetic structure with most other populations. We further predict that populations of anthropogenic origin share alleles with geographically distant wild populations.

## 2 MATERIALS AND METHODS

### 2.1 Study species

*Asimina triloba* (L.) Dunal (Annonaceae) is the most widespread member of the genus, occurring in 26 states in the eastern United States, ranging from Texas and Iowa to the eastern seaboard and north into southern Ontario, Canada (Little, 1977). This understory tree, with trunks ≤20 cm diameter at breast height (DBH; Wyatt, personal observation), generally occurs at elevations <350 m above sea level, primarily in mesic, alluvial forests along rivers and creeks (Freeman & Hulbert, 1985; Murphy, 2001; Pomper et al., 2010). Vegetative spreading occurs via root‐suckers (Keener & Kuhns, 1998), resulting in dense patches and genets that persist for a long time (Hosaka et al., 2005). While there are no reports of longevity of individual ramets or genets, we documented 32 annual growth rings in one stem from Georgia with a DBH of 11.4 cm.

Flowers, which are strongly protogynous (Lagrange & Tramer, 1985), emerge in the spring after the last frost, and flowering on a given stem continues for approximately a month (Wyatt, personal observation). Flowering is also highly synchronous (Lagrange & Tramer, 1985). *Asimina triloba* pollinators are weak flying insects such as flies (including *Drosophila* spp.), beetles, and small Lepidoptera (Goodrich et al., 2006; Robertson, 1928; Willson & Schemske, 1980). Fruit set tends to be low (Lagrange & Tramer, 1985; Willson & Schemske, 1980), with only 0.4% of flowers and 15% of stems producing fruit in a central Illinois population (Willson & Schemske, 1980). Willson and Schemske (1980) found that fruit set was subject to pollen‐limitation as well as resource limitation related to the size of the fruits. *Asimina triloba* produces the largest edible fruits (≤1 kg; Darrow, 1975) of any native North American tree. Lagrange and Tramer (1985) also report fruit set relative to the number of flowers ranging from 0.5% to 3.4% in Tennessee and 0% in Toledo, Ohio.

Natural dispersal of *A. triloba* is likely by hydrochory and zoochory. Fruits float and are potentially dispersed along waterways (Bowden & Miller, 1951; Peterson, 1991; Wyatt, obs. pers.). The fruit is consumed by various animals with seeds reported in the scat of raccoons (Murphy, 2001; Willson, 1993; Willson & Schemske, 1980; Yeager & Elder, 1945), opossums, red fox (Murphy, 2001; Willson, 1993), and coyotes (Cypher & Cypher, 1999; Wyatt, personal observation) suggesting that these animals may mediate seed dispersal. There are reports of black bear in Virginia and North Carolina eating the fruit but seeds have not been documented in scat (Willson, 1993). Turkeys also consume the fruits (Murphy, 2001) although the seeds are digested and destroyed in the process (Wyatt, personal observation). Deer are unlikely vectors as there is no evidence that they consume the fruit other than in captivity (Murphy, 2001; Wyatt, personal observation). It is believed that historically the fruit was primarily consumed by megafauna that are now extinct or extirpated from their former range (Janzen & Martin, 1982; Poor, 1984) and that these megafauna may have mediated long‐distance dispersal of pawpaw. Mammoths (*Mammuthus columbi*), tapirs (*Tapirus veroensis*), bison (*Bison bison*), horses (*Equus* sp.), mastodons (*Mammut americanum*), and ground sloths (*Paramylodon harlani*, two species of *Eremotherium*, and three *Megalonyx* spp.) were present in Southeastern North America (Fields et al., 2012; Janzen & Martin, 1982; Poor, 1984) as recently as 12,700 YBP (Perrotti, 2018). Evidence suggests that horses and mastodons, for example, underwent annual migrations in Southeastern North America in excess of 150 km (Hoppe & Koch, 2007). Bison also migrated extensively before their numbers were decimated (Kauffman et al., 2021).

### 2.2 Sampling and genotyping

**FIGURE 1:**
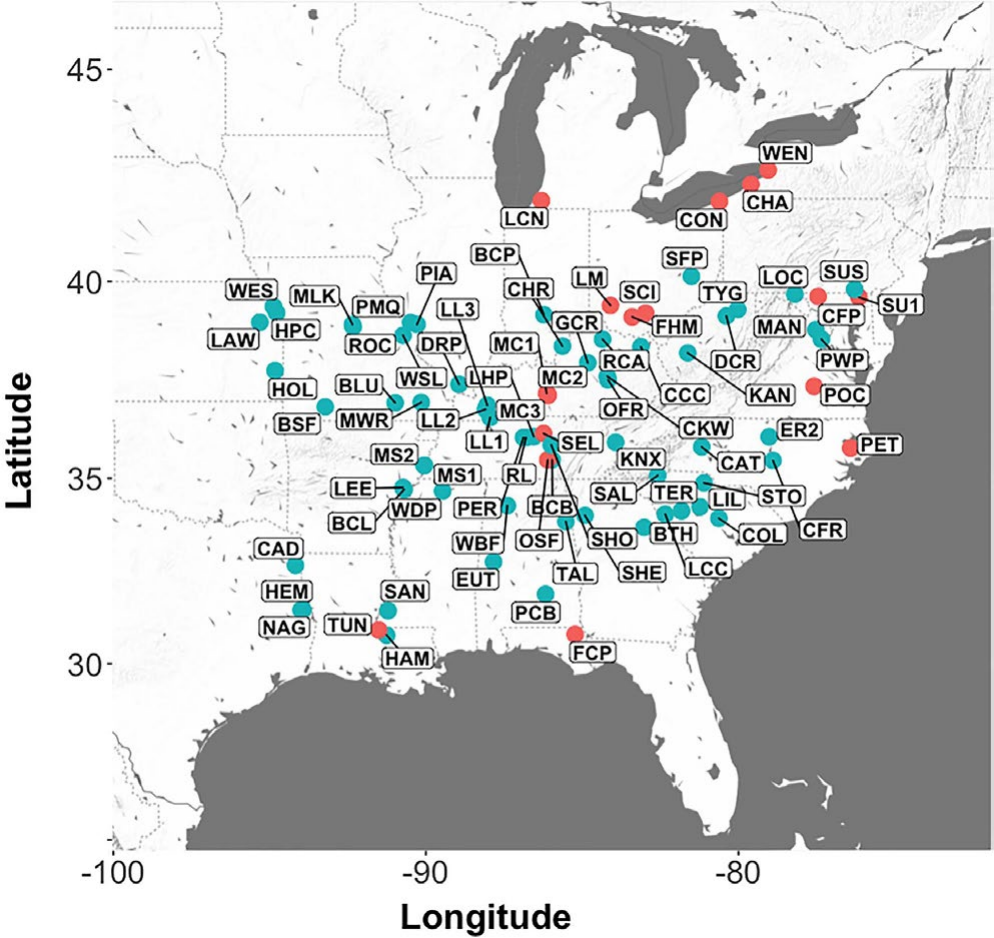
Map of 20 putative anthropogenic populations (red circles) and 62 wild populations (blue circles) of *Asimina triloba* in the eastern United States

Leaf tissue samples were collected from 82 mapped populations of *A*. *triloba* distributed throughout the species’ range (Tables 1 and 2; Table S1; Figure 1). Populations were separated from one another by a mean of 681.3 km (range = 0.2 to 1,845.8 km). Twenty populations were classified as of putative anthropogenic origin based on their location <1 km from documented pre‐Columbian habitations (i.e., villages or mound sites). We selected this objective criterion based on Muñoz et al. (2014) who indicate that impacts on plants by indigenous North Americans were localized around settlements as well as along travel corridors. Proximity to pre‐Columbian settlements increases the likelihood that populations are anthropogenic, but anthropogenic origins become less probable with increasing distance from former settlements. Six of the 20 putative anthropogenic populations (LM1, LM2, LM3, MC1, MC2, and MC3) are located near archeological sites where seeds or carbonized remains of *A*. *triloba* have been found (Keener & Kuhns, 1998; Yarnell & Watson, 1966). Sixty‐two populations were designated as “wild” as there are no known indigenous sites or trade routes located within 1 km of these populations. We compared the elevation of putative anthropogenic and wild populations and tested for significance with a two‐sample *t* test assuming equal variances.

**TABLE 1:** Locations and elevation of twenty *Asimina triloba* populations of putative anthropogenic origin, with the pre‐Columbian culture/period of nearby archeological evidence indicated. Populations are arranged in order of ascending latitude. Mean elevation is 194.6 m (*SD* = 83.6)

Pop	Location	Latitude	Longitude	Elevation (m)	Culture or period	Reference
FCP	Florida Caverns State Park, FL	30.810	−85.227	135	Fort Walton	Gardner (1966)
TUN	Tunica Hills WMA, LA	30.930	−91.514	91	Tunica	Perrault et al. (2008)
OSF	Old Stone Fort State Park, TN	35.485	−86.103	314	Middle Woodland	Yerka (2010)
PET	Pettigrew State Park, NC	35.793	−76.413	11	Late Woodland	Phelps (1983)
SEL	Sellars Farm Archeological Site, TN	36.169	−86.236	191	Mississippian	Butler (1981)
MC3	Mammoth Cave, KY	37.148	−86.093	248	Woodland	Yarnell and Watson (1966)
MC2	Mammoth Cave, KY	37.178	−86.108	164	Woodland	Yarnell and Watson (1966)
MC1	Mammoth Cave, KY	37.204	−86.139	245	Woodland	Yarnell and Watson (1966)
POC	Pocahontas State Park, VA	37.381	−77.583	87	Powhatan	Higgins et al. (1995); Kandle et al. (1996)
FHM	Fort Hill Memorial, OH	39.123	−83.401	288	Hopewell	Prufer (1964)
SCI	Scioto Trail State Park, OH	39.216	−82.974	309	Middle Woodland	Carr (2008)
LM3	Little Miami River, OH	39.409	−84.100	203	Fort Ancient	Connolly (2004)
LM2	Little Miami River, OH	39.410	−84.101	204	Fort Ancient	Connolly (2004)
LM1	Little Miami River, OH	39.410	−84.099	206	Fort Ancient	Connolly (2004)
SU1	Susquehanna State Park, MD	39.610	−76.147	74	Late Woodland	MacNamara (1982)
CFP	Cunningham Falls State Park, MD	39.633	−77.456	313	Late Woodland	Bedell et al. (2011)
CON	Conneaut, OH	41.933	−80.611	221	Woodland	Wallace (1952); Brose (1976)
LCN	Love Creek Nature Center, MI	41.951	−86.306	225	Late Woodland	Hinsdale (1931); Lovis (1974)
CHA	Chautauqua Creek, NY	42.339	−79.604	186	Late Woodland	Wallace (1952); Bennett (1986)
WEN	Wendt County Beach Park, NY	42.679	−79.052	176	Late Woodland	Bennett (1986)

Leaf samples were collected from a mean of 28.1 individuals (range = 5–50) from each anthropogenic population and a mean of 29.6 individuals (range = 10–62) from each wild population (Table 2). Samples were taken from mature (≥3 m in height) stems separated by ≥10 m when possible to avoid sampling multiple ramets belonging to the same genet. Samples were snap‐frozen in liquid nitrogen and transported to the University of Georgia for genetic analyses.

Genomic DNA was extracted from frozen leaf tissue (~0.05 g) using a modified CTAB protocol (Doyle & Doyle, 1987), and DNA quality and quantity were evaluated using an ND‐1000 NanoDrop® spectrophotometer. Nine nuclear microsatellite loci (Lu et al., 2011; Table S2) were PCR amplified using a 3‐primer protocol whereby a CAG‐tag sequence (Hauswaldt & Glenn, 2003) was added to the 5’ end of one primer and a third fluorescently labeled (FAM, HEX, or NED) primer identical to the CAG‐tag was included. The 12.5 µl reaction volumes contained 3.25 µl molecular grade ddH2O, 2.5 µl 5× One Taq^®^ standard reaction buffer (New England Biolabs, Ipswich, MA), 0.35 µl 25 mM MgCl solution (Sigma‐Aldrich), 1.25 µl 10× (1 mg/ml) bovine serum albumin (Thermo‐Fisher), 1 µl 2.5 mM dNTP mix (New England Biolabs, Ipswich, MA), 1 µl primer mix (0.5 µM CAG‐labeled primer and 5 µM unlabeled primer), 0.45 µl 10 µM universal dye‐labeled primer, 0.2 µl One Taq^®^ Hot Start DNA polymerase (New England Biolabs, Ipswich, MA), and 2.5 µl of diluted template DNA (20 ng/µl).

Presence of null alleles was evaluated in Genepop v. 4.2 using Brookfield's method (Brookfield, 1996; Raymond & Rousset, 1995). Because *A*. *triloba* propagates vegetatively, we tested whether duplicate multilocus genotypes (MLGs) within populations represented clones. To estimate the likelihood of two individuals within a population having identical MLGs by chance, we calculated the probability of identity (PI) for each population which reflects the number of loci, allele frequencies, and sample size. The multilocus probability of identity (PI*_m_*) was calculated as PI*_m_* = Π_s_ (PI_s_), where

$${\text{PI}}_{s}=\sum_{i}p_{i}^{4}+\sum_{i}\sum_{j>1}{\left(2p_{i}p_{j}\right)}^{2}$$

and *p_i_* and *p_j_* are the frequencies of the *i*th and *j*th alleles at a locus, respectively (Gonzales et al., 2008; Paetkau & Strobeck, 1994). The PI was adjusted for sample size (*N*) by PI = *1* – [(1 – PI*_m_*)*^N^*] (Parker et al., 1998). Duplicate MLGs that represented clones were removed, leaving one individual per genet for subsequent analyses.

The number of MLGs and the number of MLGs adjusted for population sample size (sMLG; i.e., rarefaction) were calculated using Fstat (Goudet, 1995, 2001). For the rarefaction analysis, all populations were treated as consisting of 10 individuals to reflect the number of individuals in the smallest population after SEL_A_ (*N* = 5), LM2_A_ (*N* = 6), and STO_W_ (*N* = 9) were omitted. Genetic diversity statistics were estimated separately for putative anthropogenic populations and wild populations using GenAlEx v. 6.51b2 (Peakall & Smouse, 2006, 2012). Measures of genetic diversity were percent of polymorphic loci (*P*), total number of alleles (*A*_T_), effective number of alleles (*A*_E_), number of private alleles (*P*_A_; an allele found in a single population), rare alleles (*A_RARE_*; alleles in <10% of all populations), observed heterozygosity (*H*_O_), expected heterozygosity (*H*_E_), and the inbreeding coefficient (*F*_IS_). For all genetic diversity statistics, we used a two‐sample *t* test assuming equal variances to assess whether there were significant differences between anthropogenic and wild populations. Population values were averaged across populations as well as pooled. Observed heterozygosity (*H*_O_) and Hardy–Weinberg expected heterozygosity (*H*_E_) were compared for each locus in each population, using Wright's inbreeding coefficient (*F*_IS_; Wright, 1922, 1951). Deviations from Hardy–Weinberg expectations were tested for significance using chi‐square (Li & Horvitz, 1953). A Bonferroni correction for multiple comparisons was applied using FSTAT (Goudet, 1995, 2001). Correlations between MLGs and *F*_IS_ values, as well as between latitude and *F*_IS_ values, were tested for significance using linear regression analyses.

Genetic structure among populations was estimated using Nei’s (1973) *G*_ST_. Pairwise *G*_ST_ values were calculated for all possible pairs of populations in GenAlEx.v. 6.5.1b2 (Peakall & Smouse, 2006, 2012). A two‐sample *t* test assuming equal variances was used to test for significant differences between mean pairwise *G*_ST_ values and mean pairwise geographic distances among anthropogenic populations versus wild populations.

For the nine putative anthropogenic populations located at, or within, the boundary of the Wisconsin glaciation we used a two‐sample *t* test assuming equal variances to assess significance between (a) pairwise *G*_ST_ values among the nine populations versus pairwise *G*_ST_ values between these populations and the wild populations with which they were most genetically similar, and (b) pairwise geographic distances of putative anthropogenic populations located at or within the boundary of the Wisconsin glaciation and the wild populations with which they shared their lowest *G*_ST_ values.

Isolation by distance (IBD; Wright, 1943) was estimated using linear regression analysis of *G*_ST_ / (1 − *G*_ST_) against log geographic distance (Rousset, 1997) for all 82 populations as well as separately for anthropogenic and wild populations. Significance was tested using a Mantel test in GenAlEx.v. 6.5.1b2 (Peakall & Smouse, 2006, 2012).

structure v. 2.3.4 (Pritchard et al., 2000), a Bayesian clustering approach, was used to estimate levels of genetic admixture among all 82 populations and the number of genetically distinct clusters (*K*). Levels of admixture and *K* were also estimated separately for putative anthropogenic populations as a part of the present study and wild populations as reported by Wyatt et al. (2021). Ten independent simulations at each *K*‐value from 1 to 20 were run, using a burn‐in of 500,000 repetitions and a run length of 1,000,000 Markov chain Monte Carlo (MCMC) iterations. The admixture model was chosen to infer alpha (α). We also employed a model based on correlated allele frequencies with no a priori assumptions regarding sampling locations. The Evanno et al. (2005) method was used to determine the optimal number of genetic clusters (*K*) in Structure Harvester (Earl & vonHoldt, 2012).

The occurrence of rare alleles in <10% of all wild and putative anthropogenic populations (i.e., alleles present in 2–8 populations) was used to more closely investigate relationships between putative anthropogenic and wild populations. A two‐sample *t* test assuming equal variances was used to assess whether the number of rare alleles in anthropogenic versus wild populations differed significantly. We also used linear regression analyses to test whether there was a significant relationship between latitude and the number of rare alleles in anthropogenic and wild populations.

We were particularly interested in examining rare alleles shared by putative anthropogenic and wild populations. We estimated pairwise *G*_ST_ values and pairwise geographic distances of all wild populations (W), pairs of wild populations sharing rare alleles (W_RA_), wild and anthropogenic populations sharing rare alleles (W – A)_RA_, anthropogenic populations in areas not formerly glaciated and wild populations sharing rare alleles (A_NG_ – W)_RA_, as well as anthropogenic populations in formerly glaciated areas and wild populations sharing rare alleles (A_G_ – W)_RA_. We used a two‐sample *t* test assuming equal variances to test for significance between pairwise *G*_ST_ values and geographic distances of (a) wild populations (W) versus wild populations that share rare alleles (W_RA_), (b) wild populations (W) versus wild and anthropogenic populations that share rare alleles ((W – A)_RA_), (c) wild populations that share rare alleles (W_RA_) versus wild and anthropogenic populations that share rare alleles ((W – A)_RA_), and (d) (A_NG_ – W)_RA_ versus (A_G_ – W)_RA_.

### 2.3 Fruit set

To better evaluate whether populations of putative anthropogenic origin and populations at the edge of the species range exist beyond or at the edge of their ecological limit, we collected fruit set data. For a species to persist in a particular landscape, individuals must be able to survive and reproduce. Fruit set was recorded for each population between May and August, although primarily in July and August, after flowering had ended at which point rapidly enlarging ovaries are visually conspicuous. Because of the large number of populations and their widespread distribution, data were collected over the course of three summers with each population surveyed once. Developing fruits occurring on all stems sampled for genotyping were counted. The stage of fruit maturation was not recorded as it was impossible to visit all populations within a sufficiently small window of time to render comparable data.

For each population, the number of fruits/stem and fruits/MLG were calculated and a two‐sample *t* test assuming equal variances was used to assess whether anthropogenic and wild populations differed significantly. We used linear regression analysis separately for anthropogenic and wild populations to test correlations between (a) the number of MLGs and the number of fruits produced, (b) latitude and the number of fruits/stem, and (c) latitude and the number fruits/MLG.

## 3 RESULTS

Ten (12%) of the 82 populations are in landscapes within, or at, the boundary of the Wisconsin glaciation maximum. Of these, nine are anthropogenic (WEN_A_, CHA_A_, LCN_A_, CON_A_, LM1_A_, LM2_A_, LM3_A_, SCI_A_, and FHM_A_) and one (BCP_W_) is wild. Thus, 45% of anthropogenic populations and 2% of wild populations occur in landscapes that were colonized after retreat of the LGM. The mean elevation of putative anthropogenic populations (194.6 ± 83.6 m) exceeded the mean for wild populations (178.8 ± 94.3 m), but the difference was nonsignificant (*p* = .253; Tables 1; Table S1).

### 3.1 Genetic diversity

Null allele frequencies were nonsignificant for all loci and populations (*p* = .05). Mean probability of identity (PI) for anthropogenic and wild populations was 1.46 × 10^–4^ and 1.15 × 10^–3^, respectively (Table S3), indicating a high likelihood that identical MLGs within populations represent clones. Despite efforts to avoid sampling multiple ramets per genet, identical MLGs were present in all but three wild populations (BTH_W_, LIL_W_, and LL3_W_). There were no MLGs shared by individuals in different populations. Although mean sample sizes for anthropogenic and wild populations were 27.3 and 29.4, respectively, six anthropogenic (30%) and eight wild (13%) populations had a single MLG.

The mean number of MLGs was significantly lower for anthropogenic than wild populations (5.3 and 10.1, respectively; *p* = .011; Table 2; Figure S1). When adjusted for sample size, the mean number of sMLGs was also significantly lower for anthropogenic populations (4.6 and 7.0, respectively; *p* = .029; Table 2).

A total of 160 alleles occurred across all 82 populations (Table 2); however, the mean was significantly lower in anthropogenic (24.6) than wild (32.5) populations (*p* = .010). A total of 34 private alleles occurred in four anthropogenic (20%) and 20 wild populations (32%). Anthropogenic populations of *A*. *triloba* had less genetic diversity than wild populations for all population genetic statistics except observed heterozygosity, although the differences were significant only for the number of MLGs (5.3 vs. 10.1; *p* = .011), sMLGs (4.6 vs. 7.0; *p* = .029), and *A*_T_ (24.6 vs. 32.5: *p* = .010). Other diversity statistics for anthropogenic versus wild populations were *P* (77.9 vs. 84.7; *p* = .198), *A*_E_ (2.2 vs. 2.6; *p* = .056), *P*_A_ (0.35 vs. 0.44; *p* = .948), *A*_RARE_ (1.9 vs. 2.9; *p* = .071)), and *H*_E_ (0.438 vs. 0.496; *p* = .114). Anthropogenic populations had a higher mean *H*_O_ (0.576) than wild populations (0.530; *p* = .199), although the difference was not significant, and a significantly lower mean inbreeding coefficient (*F*_IS_ = −0.413 vs. −0.153; *p* = .032), indicative of an excess of heterozygosity relative to Hardy–Weinberg expectations (HWE; Table 2). *F*_IS_ values were highly significant for 50% of anthropogenic populations (*p* < .005) and significant for 19% wild populations (*p* < .05; Table 2). For nearly all populations with an inbreeding coefficient that differed significantly from HWE, there was excess heterozygosity. There was a significant positive correlation between MLGs and *F*_IS_ values in anthropogenic (*r* = 0.640; *p* = .001) and wild populations (*r* = 0.570; *p* = 6.50 × 10^–7^). *F*_IS_ values also decreased significantly with increasing latitude in anthropogenic populations (*r* = −0.599; *p* = .005) but the relationship was nonsignificant in wild populations (*r* = −0.192; *p* = .134).

**TABLE 2:** Summary statistics of *A*. *triloba* nuclear genetic diversity in 20 putative anthropogenic and 62 wild populations arranged in order of ascending latitude. Data for wild populations are from Wyatt et al. (2021)

Population	*N*	MLG	sMLG	*P*	*A*_T_	*A*_E_	*P*_A_	*A*_RARE_	*H*_O_	*H*_E_	*F*_IS_
Anthropogenic											
FCP	35	3	3.0	44	17	1.8	2	4	0.185	0.290	0.348^¥^
TUN	21	7	6.1	100	45	3.9	2	7	0.651	0.668	0.044
OSF	35	3	3.0	100	30	2.9	—	4	0.630	0.537	−0.179
PET	24	22	15.1	89	33	2.3	—	2	0.479	0.495	0.051
SEL	5	2	—	78	22	2.3	—	2	0.722	0.472	−0.562
MC3	12	1	1.0	44	13	1.4	—	1	0.444	0.222	−1.000^¥^
MC2	18	10	9.6	100	36	2.5	—	1	0.658	0.541	−0.194
MC1	16	7	6.9	100	33	2.7	—	4	0.501	0.532	0.098
POC	48	6	3.7	67	21	2.0	—	—	0.537	0.403	−0.400^¥^
FHM	46	6	3.7	100	29	2.4	2	—	0.491	0.545	0.058
SCI	24	1	1.0	67	15	1.7	1	1	0.667	0.333	−1.000^¥^
LM3	14	3	3.4	78	20	2.1	—	—	0.593	0.444	−0.383
LM1	17	14	12.0	100	43	3.3	—	2	0.583	0.632	0.125
LM2	6	1	—	67	15	1.7	—	2	0.667	0.333	−1.000^¥^
SU1	33	11	6.7	89	39	3.1	—	3	0.606	0.559	−0.111
CFP	48	1	1.0	56	13	1.4	—	1	0.556	0.278	−1.000^¥^
CON	30	2	1.9	89	24	2.4	—	1	0.778	0.528	−0.467^¥^
LCN	48	4	2.5	89	18	1.8	—	3	0.778	0.448	−0.690^¥^
CHA	38	1	1.0	33	11	1.2	—	—	0.333	0.167	−1.000^¥^
WEN	10	1	1.0	67	15	1.7	—	—	0.667	0.333	−1.000^¥^
Mean	27.3	5.3	4.59	77.9	24.6	2.24	0.35	1.9	0.576	0.438	−0.413
*SD*	13.6	5.5	4.11	21.4	10.6	0.70	0.50	1.7	0.145	0.138	0.467
Pooled	546	106	—	100	98	5.69	7	—	0.591	0.709	0.170
Wild											
HAM	47	2	1.9	100	28	2.2	—	3	0.444	0.528	0.170
SAN	21	1	1.0	78	16	1.8	1	2	0.778	0.389	−1.000^¥^
NAG	29	3	2.6	78	20	3.1	—	1	0.667	0.586	−0.158
HEM	40	5	2.6	89	26	2.8	—	3	0.578	0.544	−0.016
PCB	48	14	8.0	89	29	1.9	—	6	0.397	0.391	0.013
CAD	36	21	8.0	100	31	3.3	1	2	0.633	0.657	0.085
EUT	33	32	16.5	100	57	3.9	—	13	0.463	0.650	0.231
BTH	36	36	17.3	100	54	3.4	2	8	0.620	0.665	0.066^¥^
TAL	24	3	2.7	89	25	2.2	—	1	0.611	0.480	−0.266
COL	48	18	8.2	78	29	1.9	—	2	0.394	0.397	−0.015
SHE	24	5	4.2	89	26	2.3	—	3	0.511	0.479	−0.045
LCC	24	23	15.8	89	38	2.8	—	4	0.476	0.555	0.147
TER	33	27	14.7	89	41	2.9	—	5	0.529	0.579	0.081
LIL	24	24	14.9	89	44	2.7	—	4	0.452	0.562	0.194
WBF	43	22	13.1	89	53	3.5	—	13	0.578	0.623	0.082
WDP	27	3	2.6	89	26	2.5	1	4	0.444	0.531	0.192
BCL	48	12	10.6	100	69	2.7	1	5	0.503	0.573	0.125
LEE	24	12	9.8	100	56	4.1	—	8	0.643	0.703	0.096
STO	9	5	—	89	25	2.3	—	1	0.454	0.498	0.129
SAL	30	14	9.9	100	43	3.0	—	1	0.563	0.612	0.030
MS1	24	4	3.7	78	18	3.5	—	—	0.611	0.674	0.072
MS2	23	1	1.0	67	15	0.8	1	—	0.111	0.056	−1.000^¥^
CFR	20	5	4.1	89	30	2.5	1	1	0.689	0.550	−0.238
BCB	25	8	6.4	78	21	1.9	—	3	0.597	0.404	−0.367*
CAT	16	1	1.0	67	14	1.7	—	—	0.667	0.333	−1.000^¥^
SHO	24	16	12.2	89	49	3.4	—	6	0.596	0.566	−0.062
KNX	25	3	2.7	100	31	2.8	2	2	0.537	0.600	0.133
RL	27	10	8.7	100	44	3.6	2	4	0.681	0.639	−0.064
PER	46	4	3.3	100	37	3.4	—	—	0.583	0.642	0.044
ER2	10	4	4.0	67	17	1.7	—	1	0.500	0.326	−0.527
LHP	20	4	3.5	89	22	1.9	—	2	0.611	0.406	−0.442*
LL1	25	1	1.0	56	14	1.6	—	1	0.556	0.278	−1.000^¥^
LL2	25	7	6.2	78	38	3.4	—	2	0.613	0.580	−0.055
BSF	23	21	14.9	89	43	3.1	—	5	0.546	0.543	−0.010
LL3	24	24	16.0	78	51	3.7	1	8	0.495	0.577	0.141
BLU	25	9	7.32	78	28	2.5	1	1	0.460	0.506	0.062
MWR	25	14	10.3	78	38	2.3	1	4	0.524	0.442	−0.092
DRP	23	13	10.3	100	41	3.3	2	7	0.562	0.599	0.070
OFR	33	7	5.7	89	36	2.9	—	5	0.550	0.512	−0.082
CKW	22	13	10.0	100	40	2.5	4	2	0.523	0.526	−0.024
HOL	10	9	9.0	89	34	2.9	—	2	0.545	0.495	−0.098
GCR	48	5	3.5	78	26	2.4	1	1	0.356	0.471	0.316^§^
KAN	43	10	7.7	89	43	3.4	—	1	0.559	0.599	0.090
CCC	26	12	9.5	89	43	3.2	—	5	0.477	0.614	0.222
CHR	30	19	12.5	89	45	3.2	—	4	0.580	0.598	0.030
PWP	48	27	14.3	100	56	3.6	—	6	0.540	0.670	0.169
RCA	24	16	11.6	100	49	3.8	1	2	0.490	0.645	0.273
WSL	11	9	9.0	100	40	3.5	—	6	0.600	0.647	0.078
MAN	48	1	1.0	33	10	1.1	—	—	0.333	0.167	−1.000^¥^
ROC	25	1	1.0	56	14	1.6	—	—	0.556	0.278	−1.000^¥^
MLK	24	2	1.9	67	18	1.8	1	—	0.611	0.375	−0.622^¥^
PIA	14	1	1.0	44	15	1.4	1	1	0.444	0.222	−1.000^¥^
PMQ	59	4	3.5	89	26	2.2	—	1	0.583	0.441	−0.313^§^
LAW	30	12	7.8	89	29	1.9	—	2	0.492	0.392	−0.185
DCR	15	1	1.0	44	13	1.4	—	—	0.444	0.222	−1.000^¥^
BCP	48	2	2.0	67	17	1.7	1	—	0.444	0.292	−0.547^¥^
HPC	25	15	10.6	89	31	2.1	–	—	0.467	0.469	−0.020
TYG	48	2	2.0	89	20	2.0	—	1	0.722	0.444	−0.600^¥^
WES	22	9	7.3	100	45	3.1	—	5	0.537	0.590	0.078
LOC	38	3	2.2	56	16	1.6	—	—	0.278	0.264	−0.134
SUS	30	8	6.7	100	37	3.0	—	2	0.602	0.603	0.088
SFP	25	7	6.0	100	27	2.1	1	—	0.456	0.482	0.014
Mean	29.4	10.1	7.01	84.7	32.5	2.59	0.44	2.9	0.530	0.496	−0.153
*SD*	11.5	8.6	4.83	15.7	13.5	0.77	0.75	2.9	0.108	0.141	0.387
Pooled	1824	626	—	100	153	6.52	27	—	0.553	0.765	0.273
Species mean	28.9	8.9	6.46	83.0	30.6	2.50	0.41	2.7	0.541	0.482	0.216
Species pooled	2,370	732	—	100	160	6.60	34	54	0.559	0.760	0.260

Notes

*N* = sample size, MLG = number of multilocus genotypes, sMLG = number of multilocus genotypes adjusted for sample sizes after the three smallest populations were omitted (LM2_A_, SEL_A,_ and STO_W_), *P* = percent of polymorphic loci, *A*_T_ = total number of alleles, *A*_E_ = effective number of alleles, *P*_A_ = number of private alleles, *A*_RARE_ = number of rare alleles (<10% of all anthropogenic and wild populations), *H*_O_ = observed heterozygosity, *H*_E_ = expected heterozygosity, *F*_IS_ = inbreeding coefficient.

Significant *F*_IS_ values are indicated with * (*p* < .05), § (*p* < .01) and ¥ (*p* < .005).

### 3.2 Genetic structure

Genetic structure (*G*_ST_) among anthropogenic and wild populations was 0.388 and 0.345, respectively. There was significant IBD among all 82 populations (*r* = 0.143; (P(rxy‐rand > = rxydata) = 0.010) and among the 62 wild populations (*r* = 0.158; (P(rxy‐rand > = rxydata) = 0.020), but among anthropogenic populations there was an absence of significant IBD (*r* = 0.068; (P(rxy‐rand > = rxydata) = 0.230). Although the mean geographic distance separating pairs of anthropogenic populations was less than between wild populations (629.3 vs. 660.3 km; *p* = .125), the mean pairwise *G*_ST_ value was significantly higher for anthropogenic populations (0.265 vs. 0.228; *p* = 1.12 × 10^–7^).

The nine northernmost populations of putative anthropogenic origin occupying landscapes at, or within, the boundary of the Wisconsin glaciation maximum (CHA_A_, CON_A_, FHM_A_, LCN_A_, LM1_A_, LM2_A_, LM3_A_, SCI_A,_ and WEN_A_) had a mean pairwise *G*_ST_ = 0.289 (range = 0.071 to 0.523) and were separated from one another by a mean of 317 km (range = 0.2 to 602 km). These populations instead shared their lowest pairwise *G*_ST_ values (mean = 0.137; range = 0.037 to 0.268) with wild populations that were a mean of 865 km away (range = 579 to 1,342 km). Not only did these anthropogenic populations and their genetically closest wild populations have significantly lower pairwise *G*_ST_ values (*p* = 1.29 × 10^–4^) than these nine anthropogenic populations had with each other, but they were also significantly further apart (*p* = 1.69 × 10^–8^). These anthropogenic populations and their genetically closest wild populations also had significantly lower pairwise *G*_ST_ values (*p* = .001) and were significantly further apart (*p* = .040) than all wild populations. The one wild population at the boundary of the Wisconsin glaciation (BCP_W_) is 350 km from the population with which it was most genetically similar (*G*_ST_ = 0.161).

**FIGURE 2:**
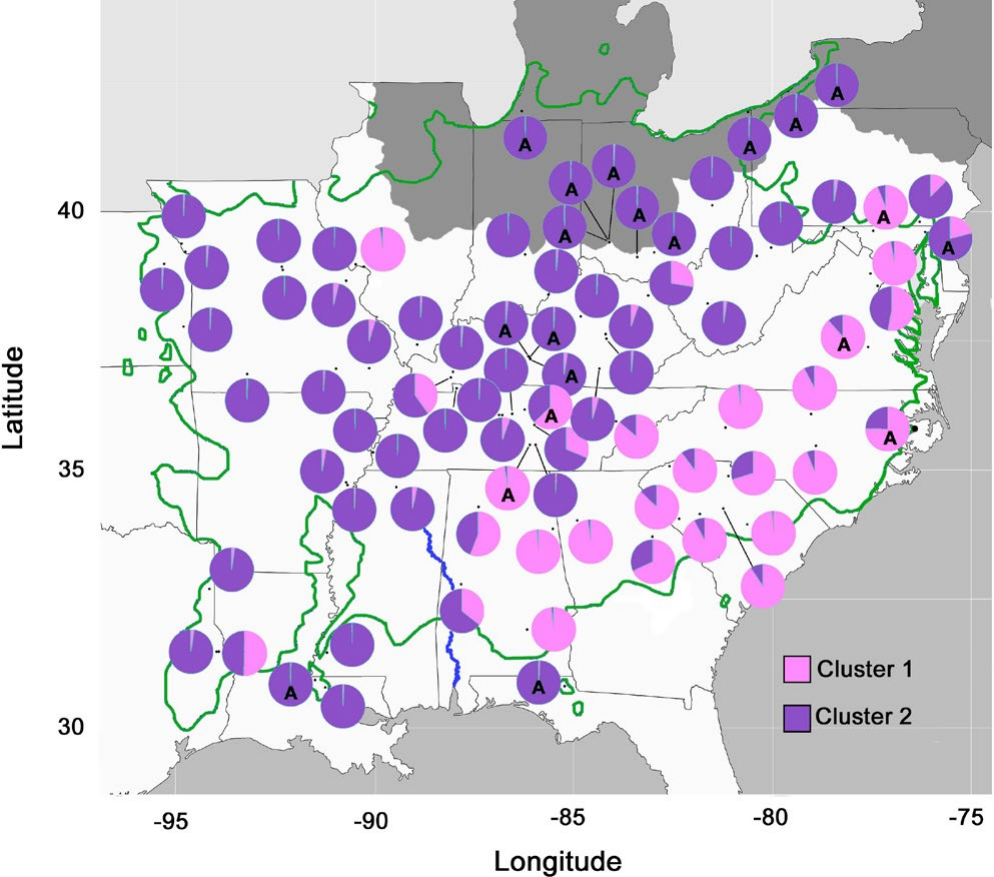
All sampled populations (*N* = 82) showing proportion of each population assigned to the two clusters identified by Structure v 2.3.4. The Tombigbee River in Alabama is indicated in blue. The reported range of *A*. *triloba* from Little (1977) is outlined in green, and the maximum extent of the Wisconsin glaciation is indicated in dark gray. A = putative anthropogenic populations. Wild populations have no designation

**FIGURE 3:**
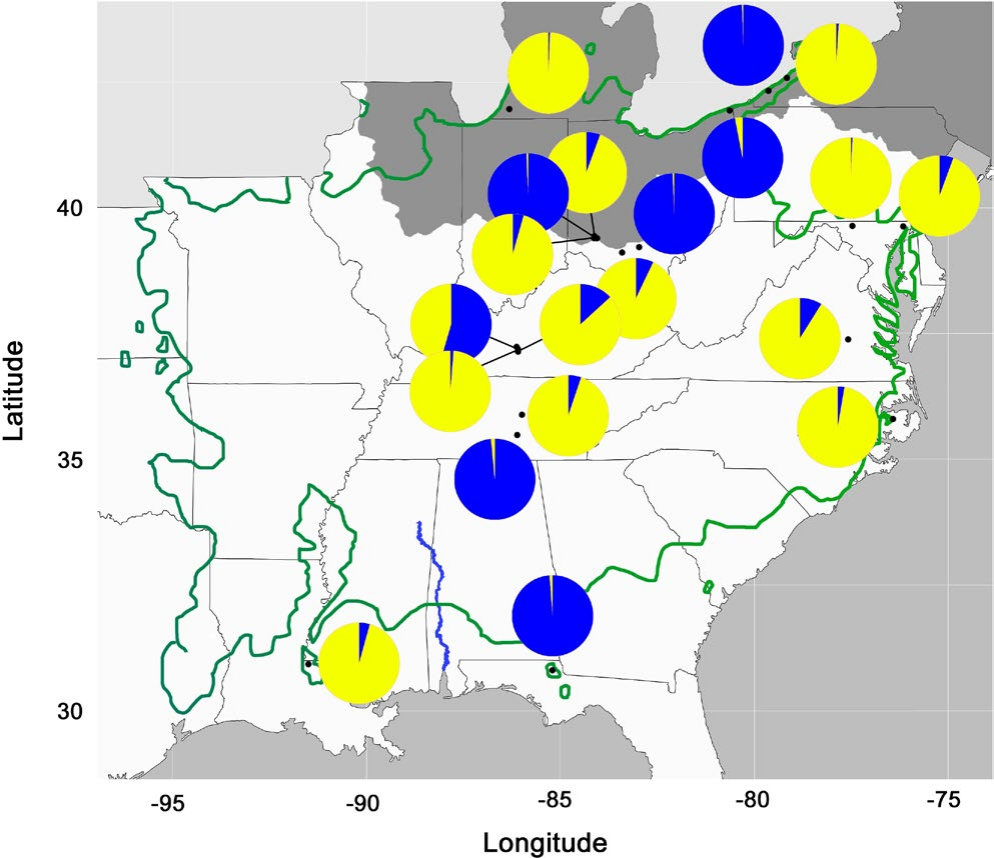
Putative anthropogenic populations of *A*. *triloba* showing the proportion of assignment to the two genetic clusters identified by Structure v 2.3.4

Simulations in Structure yielded an optimal *K* = 2 for all 82 populations, with mean Ln P(*D*) of −67,119.0 and Δ*K* of 20.6 (Table 3; Table S4; Figure 2; Figure S2). The next most likely number of clusters was *K* = 4 with mean Ln P(*D*) of −63,063.9 and Δ*K* of 8.5 (Table 3; Figures S2 and S3). Caution is required in interpretation of Structure results for all 82 populations as well as the 62 wild populations as the presence of significant IBD violates one of the underlying assumptions of Structure (Pritchard et al., 2000). Simulations for the 20 putative anthropogenic populations also found *K* = 2 to be optimal, but the clusters are not geographically distinct: populations with a higher genetic assignment to one cluster are often geographically embedded in the other cluster (Figure 3).

**TABLE 3:** Structure output for all 82 populations of *A*. *triloba* for *K* values 2 – 10, without population location data included in the models

*K*	Mean LnP(*K*)	Delta *K*
1	−70,698.6	—
2	−67,119.0	20.5702
3	−65,873.1	0.7354
4	−63,063.9	8.4830
5	−61,303.8	3.7374
6	−60,298.4	0.8305
7	−58,730.3	1.1389
8	−57,735.0	0.4290
9	−56,464.2	0.1425
10	−55,300.5	—

### 3.3 Rare alleles

**FIGURE 4:**
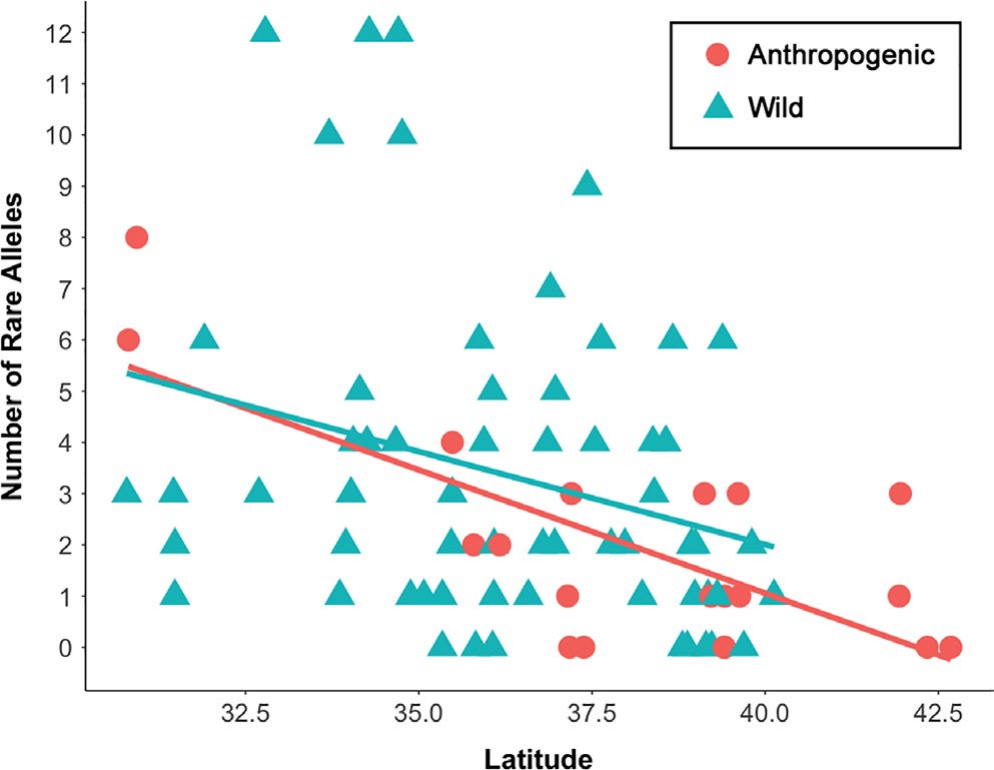
Correlation between latitude and the number of rare alleles (i.e., present in <10% of all 82 populations) for twenty anthropogenic (*r* = −0.679; *p* = .001) and 62 wild populations (*r* = −0.301; *p* = .017) of *Asimina triloba*

A total of 54 rare alleles occurred in 75% (15) of anthropogenic and 81% (50) of wild populations. Only 24 rare alleles were shared by both anthropogenic and wild populations, and no rare alleles occurred exclusively in anthropogenic populations (Figure S4). There were also fewer rare alleles in anthropogenic populations (mean = 1.9 vs. 2.9; *p* = .071). The number of rare alleles per population decreased significantly with increasing latitude for both anthropogenic (*r* = −0.679; *p* = .001) and wild populations (*r* = −0.302; *p* = .017; Figure 4).

As predicted, the pairwise *G*_ST_ values between wild populations that share rare alleles (W_RA_) were significantly smaller than for all wild populations (W; 0.166 and 0.228, respectively; *p* = 1.37 × 10^–23^) and pairwise geographic distances for W_RA_ were significantly smaller (607.1 and 660.3 km, respectively; *p* = .014; Table 4). Wild and anthropogenic populations that shared rare alleles (W ‐ A)_RA_ had a significantly higher pairwise *G*_ST_ values than W_RA_ (0.185 vs. 0.166; *p* = .012) but were also separated by significantly larger pairwise geographic distances (695.3 vs. 607.1 km; *p* = .014; Table 4). Anthropogenic populations in formerly glaciated landscapes and the wild populations with which they shared rare alleles were separated by a larger mean pairwise distance (723.1 vs. 687.7 km; *p* = 0.345) and had a significantly higher mean *G*_ST_ (0.216 vs. 0.176; *p* = 0.018) (Table 4) than anthropogenic populations in areas not formerly glaciated and wild populations that share rare alleles.

**TABLE 4:** Summary of pairwise geographic distances (km) and *G*_ST_ values between all wild populations of *A*. *triloba* (W), wild populations that share rare alleles (W_RA_), wild and anthropogenic populations that share rare alleles ((W – A)_RA_), anthropogenic populations in areas not formerly glaciated and wild populations that share rare alleles ((A_NG_ – W)_RA_), and anthropogenic populations in formerly glaciated areas and wild populations that share rare alleles ((A_G_ – W)_RA_). Statistically significant comparisons are bolded

	W	W_RA_	W	(W – A)_RA_	W_RA_	(W – A)_RA_	(A_NG_ – W)_RA_	(A_G_ – W)_RA_
Mean km	660.3	607.1	660.3	695.3	607.1	695.3	687.7	723.1
*SD*	351.4	331.8	351.4	392.3	331.8	392.2	410.5	331.7
*p*‐value	**.014**		.150		**.014**		.345	
Mean G_ST_	0.228	0.166	0.228	0.185	0.166	0.185	0.176	0.216
*SD*	0.091	0.064	0.091	0.083	0.064	0.083	0.080	0.090
*p*‐value	**1.37 × 10^−23^**		**2.71 × 10^−7^**		**.012**		**.018**	

### 3.4 Fruit Set

Fruit set occurred in fewer anthropogenic than wild populations (35% vs. 40%). Anthropogenic populations also produced fewer fruits per stem (mean = 0.10 vs. 0.29; *p* = .175) and fewer fruits per MLG than wild populations (mean = 0.40 vs. 0.47; *p* = .386; Table S5). Of the nine anthropogenic populations in formerly glaciated habitat, only two produced fruit (SCI_A_ = 1 and LCN_A_ = 1; Table S5). In wild populations, fruit set was significantly positively correlated with the number of MLGs (*r* = 0.498; *p* = 3.77 × 10^–5^), but the relationship was not significant for anthropogenic populations (*r* = 0.219; *p* = .354). In both anthropogenic and wild populations, fruit set per stem and per MLG decreased nonsignificantly (*p* > .05) with increasing latitude.

## 4 DISCUSSION

Consistent with our a priori expectations, all measures of genetic diversity indicate that putative anthropogenic populations possess less genetic variation than wild populations, with four measures differing significantly. Only observed heterozygosity was higher in anthropogenic populations, consistent with their significantly lower *F*_IS_ values. The low number of MLGs and sMLGs that characterize anthropogenic populations appear to be the most important distinguishing characteristics. Reduced genetic diversity in populations near sites historically occupied by humans has also been reported in *Agave* (Parker et al., 2007), *Spondias* (Miller & Schaal, 2006), *Phaseolus* (Bitocchi et al., 2012), *Theobroma* (Motamayor et al., 2002), and *Annona* (Larranaga et al., 2017; see also Levis et al., 2017). Reduced genetic variation is consistent with founder effects where few seeds or ramets served as colonists, introducing only a subset of the genetic variation present in source populations. It also suggests that putative anthropogenic populations of *A*. *triloba* have experienced little gene flow subsequent to their initiation.

The absence of more significant differences may relate to the unavoidably imperfect characterization of study populations. Some populations that we designated as wild may in fact be anthropogenic in origin. Particularly, suspect are populations with genetic profiles (e.g., few MLGs and sMLGs, low *A*_T_ and high *F*_IS_) similar to those of populations associated with documented pre‐Columbian settlements or mound sites. It is also possible that some populations that we designated as anthropogenic are actually wild. Incomplete documentation regarding pre‐Columbian population sites could lead to erroneous designations, and the dating of occupation of some sites as far back as 15,000–17,000 YBP (Adovasio et al., 1978; Delcourt & Delcourt, 2004; Morse & Morse, 1983; Williams et al., 2018) could mean the erosion of genetic signatures of anthropogenic origins.

Regarding the inverse relationship between genetic diversity and excess heterozygosity, wild populations of *Fagus* in Europe reveal a similar pattern (Comps et al., 2001). Excess heterozygosity appears to be correlated with asexual reproduction (Ballou et al., 2003; Birky, 1996; Bryzski & Culley, 2011; Meloni et al., 2013) which agrees with our finding of significantly higher clonality and excess heterozygosity in anthropogenic populations of *A*. *triloba* (Table 2). Excess heterozygosity may result from dominance of well‐adapted, highly heterozygous genotypes or the introduction of a few seeds from multiple genetically divergent source populations. If humans intentionally transported the fruits or seeds from wild populations, it is likely that propagules were selected on the basis of favorable traits (i.e., fruit production, fruit size, flavor, and/or fiber quality; Peattie, 1950; Peterson, 1991) possibly associated with higher heterozygosity.

The significant decline in *F*_IS_ values with increasing latitude in anthropogenic populations is also consistent with studies showing that populations at the edge of a species’ range, where they are at their ecological limit, tend to have more asexual reproduction (Billingham et al., 2003; Dorken & Eckert, 2001; Eckert, 2002; Garcia et al., 2000; Silvertown, 2008). That this inverse relationship was highly significant in anthropogenic populations but not in wild populations supports the hypothesis that the more northern populations resulted from anthropogenic dispersal into habitat at *A*. *triloba's* ecological limit. The low fruit set documented in these northern populations is consistent with low rates of gene flow and possible marginal ecological conditions for *A*. *triloba*. White (1906) also reported that flower and fruit production in *A*. *triloba* decreased with increasing latitude north of the Ohio River mouth (Cairo, Illinois) and that its vegetative growth at the northern edge of its contemporary range is beyond its fruiting limit. Murphy (2001) further mentions the reduced size of *A*. *triloba* in northern populations, although he attributes this to possible overgrazing by a deer population that has been unnaturally large since 1912 (Bartlett, 1958).

**FIGURE 5:**
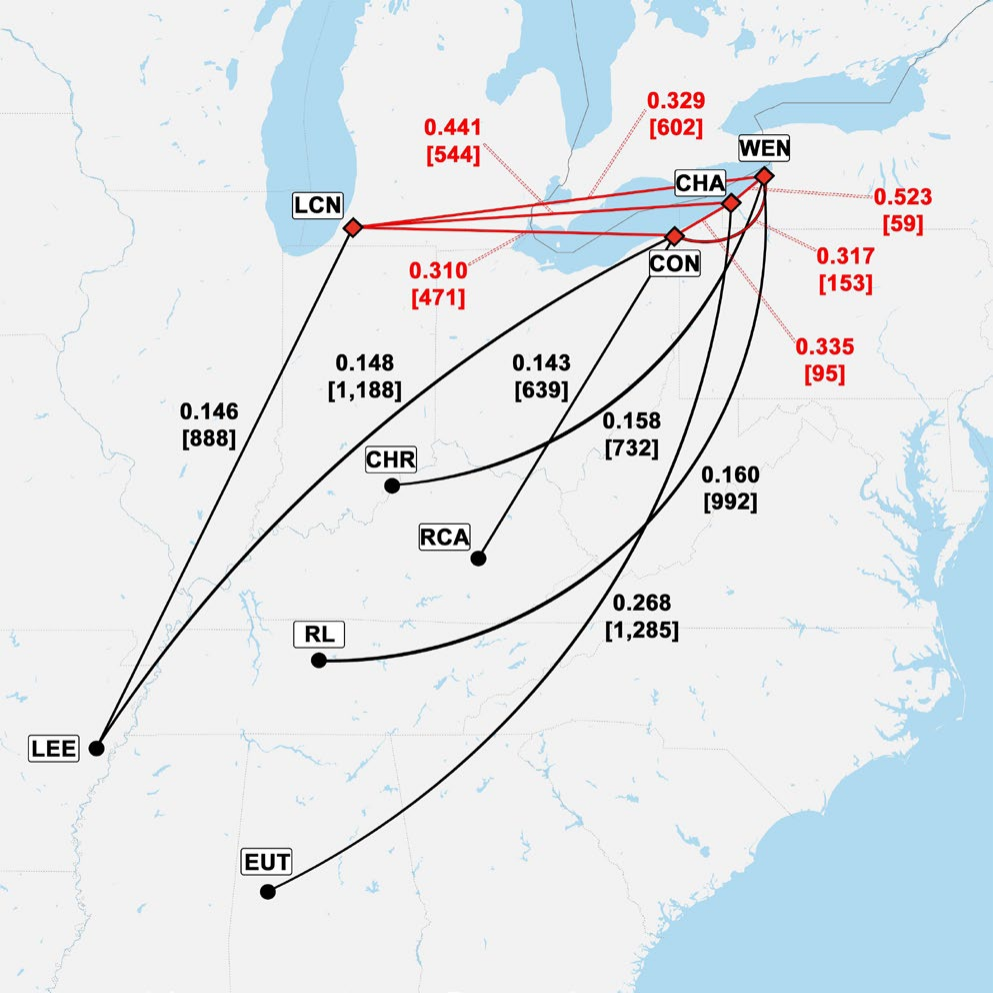
Map showing pairwise *G*_ST_ values and geographic distances in km (shown in brackets) among the four northernmost *A*. *triloba* populations (LCN, CON, CHA, and WEN), of putative anthropogenic origin, as well as between these four populations and the wild populations with which they share the lowest *G*_ST_ values. Red diamonds designate anthropogenic populations, and black circles designate wild populations. Red lines connect the anthropogenic populations while black lines connect anthropogenic and wild populations

Compelling evidence that putative anthropogenic and wild populations were shaped by different processes comes from the finding of significantly higher mean pairwise *G*_ST_ values among anthropogenic populations despite their smaller pairwise geographic distances relative to wild populations. Some of the most interesting data come from the nine northernmost anthropogenic populations that occur in formerly glaciated landscapes. Although separated from one another by a mean of 317 km, they shared their lowest pairwise *G*_ST_ values with wild populations at a mean distance of 865 km. This suggests that the wild populations with which they shared their lowest *G*_ST_ values may have served as seed sources and that subsequent to long‐distance dispersal and colonization, there has been little gene flow among these northern populations (Hewitt, 1999). The four northernmost anthropogenic populations and their most genetically similar wild populations are illustrated in Figure 5.

The finding of significant IBD in all 82 populations and all wild populations is consistent with findings for about half of angiosperms surveyed (*N* = 71; Perez et al., 2018). The absence of significant IBD in anthropogenic populations however is more noteworthy and is consistent with their hypothesized initiation by propagules from different and geographically more distant wild populations, as well as limited gene flow.

The two genetic clusters identified by Structure analysis of all 82 populations are consistent with results for wild populations only (Wyatt et al., 2021), with clusters primarily located on either side of the Appalachian Mountains and separated by an east‐west phylogeographic discontinuity along the Tombigbee drainage in Alabama (Figure 2). However, some anthropogenic populations (e.g., FCP_A_, OSF_A_, SEL_A_, and SU1_A_) are located outside the geographic boundaries of their inferred genetic cluster, which is consistent with long‐distance human‐mediated dispersal.

Alleles that occur in only a small proportion of populations can be useful for inferring patterns of dispersal. A rare allele in an anthropogenic population possessing low levels of genetic variation and located a considerable distance from the nearest wild population possessing that allele suggests long‐distance dispersal. Cases where the anthropogenic population occurs in habitat that was glaciated during the Late Wisconsin Glaciation suggest that dispersal may have been mediated by human activity as the megafauna potentially capable of such long‐distance dispersal of *A*. *triloba* went extinct ~12,000 YBP (Janzen & Martin, 1982; Perrotti, 2018; Poor, 1984). Our data show a pattern of putative anthropogenic populations occurring well north of the wild populations with which they share rare alleles. While alleles identical in size and occurring in such widely separated populations may reflect homoplasy, the low *G*_ST_ values between geographically distant anthropogenic and wild populations as well as reduced genetic diversity of putative anthropogenic populations lend support to the hypothesis that human‐mediated long‐distance dispersal was responsible for the founding of these anthropogenic populations.

Some of the most compelling evidence comes from the pairwise *G*_ST_ values and geographic distances between populations sharing rare alleles. As predicted, wild populations that share rare alleles (W_RA_) had significantly lower pairwise *G*_ST_ values and are separated by significantly smaller distances than all wild populations (W; Table 4). However, wild and anthropogenic populations that shared rare alleles, (W – A)_RA_, are separated by significantly larger distances and had significantly higher pairwise *G*_ST_ values than W_RA_ (Table 4). Anthropogenic populations in formerly glaciated landscapes and the wild populations with which they share rare alleles, (A_G_ – W)_RA_ are separated by the largest mean geographic distance investigated (Table 4).

Subsequent to extinction of North American megafauna and the LGM, the primary nonanthropogenic candidates for dispersal are waterways, raccoons, opossums, turkeys, red foxes, and coyotes. However, hydrochory cannot explain the northward migration of *A*. *triloba*. Many of the anthropogenic populations occur west of the Appalachians along the Mississippi/Ohio river drainages where the directionality of water flow is from the northeast to the southwest. While this makes hydrochory an unlikely mechanism of northward dispersal, the distribution of anthropogenic populations is consistent with known pre‐Columbian trade routes (e.g., Forsberg, 2003; Muñoz et al., 2014). Major rivers east of the Appalachians flow from west to east or northwest to southeast, also precluding the possibility of northward dispersal by hydrochory. While raccoons consume pawpaw, they are unlikely to mediate long‐distance dispersal due to their generally small home ranges of 1–3 km (maximum = 6.4 km; Stuewer, 1943; Butterfield, 1944; Feldhamer et al., 1982). Opossums are also unlikely long‐distance seed dispersers with average home ranges of 64.4 ha and 141.6 ha for females and males, respectively, and average nightly movements of 1,465 m (females) and 1,835 m (males; Ryser, 1995). Wild turkeys have annual home ranges of 150 to 550 ha but they are nonmigratory (National Wild Turkey Federation, 2021). Furthermore, turkeys that consume pawpaw appear to destroy the seeds (Wyatt, personal observation). Red foxes (*Vulpes vulpes*) have larger home ranges; however, native red foxes are a boreal species whose distribution prior to European settlement overlapped very little with the postglacial distribution of pawpaw (Kamler & Ballard, 2002). Non‐native red foxes from Europe were introduced into the eastern United States and expanded westwards in the early 1900s (Kamler & Ballard, 2002). Neither the distribution of native red foxes nor the migration of introduced red foxes are consistent with northward range expansion of *A*. *triloba*. Coyotes are the only other animals known to consume pawpaw that are capable of long‐distance movement; however, their distribution during the Pleistocene, early Holocene, pre‐Columbian period, and the early 1600s was confined to southern Mexico and Central America (Hidalgo‐Mihart et al., 2004). It is only in the last century that coyotes have expanded their range to overlap with the distribution of pawpaw (Hody & Kays, 2018). Thus, while hydrochory and zoochory have certainly contributed to *A*. *triloba* migration and range shifts, our data strongly suggest that humans played a role in long‐distance dispersal.

In summary, this study represents the first investigation into human‐mediated dispersal of a useful understory tree species in Eastern North America using genetic markers. Perhaps the most salient finding was that nearly all genetic diversity statistics are higher, although not significantly so, in wild populations than putative anthropogenic populations, except *H*_O_ which is significantly lower in wild populations. Lower genetic diversity in anthropogenic populations of *A*. *triloba* is likely associated with founder events and dispersal of seeds or ramets from wild populations to areas isolated and/or less ecologically suitable. The high levels of genetic structure observed in northern anthropogenic populations suggest independent dispersal and colonization events from southern source populations and low levels of subsequent gene flow between these populations, which is consistent with the expectation of human‐mediated dispersal into northern habitats.

As we learn more about movement and trade practices of indigenous people in Eastern North America, it has become increasingly evident that they were exchanging goods over distances of 1,000 km as early as the Archaic Period (~8,000–3,000 YBP; Sanger et al., 2018). The preponderance of anthropogenic populations along major river valleys is consistent with known trade corridors and continental‐scale exchange along large mid‐continental rivers including the Mississippi and Ohio (e.g., Fowler, 1975; Smith, 2010). Given the active and widespread trade practices of indigenous peoples of Eastern North America as well as the duration of such practices, human‐mediated dispersal is a viable explanation for the patterns of genetic variation we detected.

Populations of *A*. *triloba* become very patchy and infrequent north of 40°N (Keener & Kuhns, 1998; Wyatt, personal observation). Regardless of the dispersal vector, the high *G*_ST_ values among the northernmost *A*. *triloba* populations are indicative of multiple separate introductions from genetically distinct source populations followed by historically low rates of gene flow. The low fruit set documented in these northern populations is consistent with little sexual reproduction, which may be related to the extent of clonality in these populations and self‐incompatibility (Wyatt et al., personal observation) as well as low rates of gene flow. Low gene flow in turn may result from the isolation of populations. It is also possible that these populations exist at or beyond their ecological limit, and/or that of their pollinators, such that pollination is limited and populations persist primarily through asexual reproduction. Excellent examples of this phenomenon come from *Agave delamateri* and *A*. *murpheyi* (Parker et al., 2007), which are believed to have been introduced into Arizona by pre‐Columbian peoples because of their value as a reliable source of food, fiber, and medicine. Both species are associated with indigenous settlements that were abandoned centuries ago due to a drying climate and have persisted through clonal spreading with no evidence of sexual reproduction (Parker et al., 2007). It is thought that Arizona is outside the normal range of these species, precluding sexual reproduction. While the low pawpaw fruit set we documented represents a snapshot of one reproductive season, it is consistent with the complete absence of *A*. *triloba* seeds in refuse middens from Archaic sites around Lake Erie and northward (Keener & Kuhns, 1998). Such absence is consistent with the arrival of *A*. *triloba* at these locations more recently than 3,000 YBP and/or with the persistence of these populations primarily through asexual reproduction. This work elucidates the possible origins of populations of a widespread Eastern North American tree species at the northern limit of its range. Furthermore, closer examination of populations of *A*. *triloba* that are designated as wild for genetic profiles more similar to anthropogenic populations may reveal hitherto undetected locations of pre‐Columbian settlements and trade routes, thus furthering our understanding of indigenous cultures (e.g., Parker et al., 2014).

## CONFLICT OF INTEREST

The authors declare no conflict of interest.

## AUTHOR CONTRIBUTIONS

**Graham E. Wyatt:** Conceptualization (supporting); Data curation (lead); Formal analysis (equal); Investigation (lead); Methodology (equal); Visualization (lead); Writing‐original draft (equal). **J. L. Hamrick:** Conceptualization (supporting); Methodology (equal); Supervision (supporting); Writing‐original draft (supporting); Writing‐review & editing (equal). **Dorset W. Trapnell:** Conceptualization (lead); Data curation (supporting); Formal analysis (equal); Funding acquisition (lead); Methodology (lead); Project administration (lead); Resources (lead); Supervision (lead); Writing‐original draft (equal); Writing‐review & editing (lead).

## Supporting information

Supplementary Material

## Data Availability

Genetic data are accessible at Dryad (https://doi.org/10.5061/dryad.5x69p8d3g).
