# Supplementary material for: The role of anthropogenic dispersal in shaping the distribution and genetic composition of a widespread North American tree species
Source: Ecol Evol. 2021 Jul 30;11(16):11515–32. doi: 10.1002/ece3.7944 (PMC8366864; doi:10.1002/ece3.7944)
Supplement: Supplementary file 1 — Supplementary Material [file ECE3-11-11515-s001.docx]

*Ecology and Evolution*

**Supporting Information**

**The role of anthropogenic dispersal in shaping the distribution and genetic composition of a widespread North American tree species**

Graham E. Wyatt, J.L. Hamrick, Dorset W. Trapnell

**Table S1.**  Locations and elevation of 62 wild populations of *Asimina triloba* in the United States, arranged in order of ascending latitude. Mean elevation is 178.8 m (*SD* = 94.3).

| **Population** | **County, State** | **Latitude** | **Longitude** | **Elevation (m)** |
| --- | --- | --- | --- | --- |
| HAM | West Feliciana, LA | 30.786 | -91.255 | 21 |
| SAN | Adams, MS | 31.460 | -91.211 | 55 |
| NAG | St. Augustine, TX | 31.480 | -93.995 | 89 |
| HEM | Sabine, TX | 31.482 | -93.936 | 95 |
| PCB | Pike, AL | 31.907 | -86.174 | 125 |
| CAD | Harrison, TX | 32.693 | -94.176 | 71 |
| EUT | Greene, AL | 32.785 | -87.837 | 34 |
| BTH | Oglethorpe, GA | 33.706 | -83.019 | 156 |
| TAL | Cleburne, AL | 33.859 | -85.524 | 240 |
| COL | Richland, SC | 33.947 | -80.629 | 39 |
| SHE | Paulding, GA | 34.022 | -84.911 | 281 |
| LCC | McCormick, SC | 34.054 | -82.343 | 129 |
| TER | Saluda, SC | 34.147 | -81.824 | 142 |
| LIL | Fairfield, SC | 34.253 | -81.233 | 70 |
| WBF | Winston, AL | 34.284 | -87.396 | 183 |
| WDP | Marshall, MS | 34.668 | -89.467 | 116 |
| BCL | Lee, AR | 34.706 | -90.698 | 69 |
| LEE | Lee, AR | 34.760 | -90.733 | 61 |
| STO | York, SC | 34.881 | -81.104 | 167 |
| SAL | Pickens, SC | 35.073 | -82.587 | 349 |
| MS1 | Shelby, TN | 35.340 | -90.051 | 103 |
| MS2 | Shelby, TN | 35.341 | -90.055 | 85 |
| CFR | Harnett, NC | 35.469 | -78.898 | 38 |
| BCB | Coffee, TN | 35.485 | -85.961 | 337 |
| CAT | Catawba, NC | 35.821 | -81.177 | 269 |
| SHO | Cannon, TN | 35.872 | -85.991 | 408 |
| KNX | Knox, TN | 35.941 | -83.931 | 266 |
| RL | Davidson, TN | 36.063 | -86.799 | 233 |
| PER | Davidson, TN | 36.065 | -86.880 | 226 |
| ER2 | Orange, NC | 36.083 | -79.021 | 134 |
| LHP | Davidson, TN | 36.087 | -86.551 | 173 |
| LL1 | Stewart, TN | 36.581 | -87.947 | 150 |
| LL2 | Trigg, KY | 36.792 | -88.096 | 135 |
| BSF | Christian, MO | 36.858 | -93.222 | 286 |
| LL3 | Trigg, KY | 36.900 | -88.040 | 131 |
| BLU | Carter, MO | 36.961 | -90.987 | 137 |
| RCA | Laurel, KY | 36.962 | -84.351 | 248 |
| MWR | Stoddard, MO | 36.970 | -90.151 | 105 |
| DRP | Johnson, IL | 37.430 | -88.942 | 126 |
| OFR | Jackson, KY | 37.542 | -84.174 | 259 |
| CKW | Madison, KY | 37.632 | -84.196 | 309 |
| HOL | Bourbon, KS | 37.777 | -94.827 | 252 |
| GCR | Anderson, KY | 37.975 | -84.825 | 178 |
| KAN | Kanawha, WV | 38.224 | -81.625 | 249 |
| CCC | Carter, KY | 38.379 | -83.123 | 256 |
| CHR | Clark, IN | 38.398 | -85.635 | 146 |
| PWP | Prince William, VA | 38.566 | -77.346 | 43 |
| WSL | St. Charles, MO | 38.666 | -90.732 | 151 |
| MAN | Prince William, VA | 38.805 | -77.525 | 82 |
| ROC | Boone, MO | 38.875 | -92.327 | 207 |
| MLK | Boone, MO | 38.936 | -92.351 | 195 |
| PIA | Jersey, IL | 38.939 | -90.285 | 137 |
| PMQ | Jersey, IL | 38.984 | -90.490 | 162 |
| LAW | Douglas, KS | 38.991 | -95.315 | 264 |
| DCR | Harrison, WV | 39.146 | -80.391 | 335 |
| BCP | Brown, IN | 39.175 | -86.222 | 291 |
| HPC | Platte, MO | 39.228 | -94.778 | 280 |
| TYG | Taylor, WV | 39.308 | -80.027 | 360 |
| WES | Platte, MO | 39.384 | -94.880 | 280 |
| LOC | Morgan, MD | 39.692 | -78.199 | 177 |
| SUS | Lancaster, PA | 39.808 | -76.286 | 140 |
| SFP | Guernsey, OH | 40.128 | -81.510 | 251 |

**Table S2.**  Nuclear microsatellite loci used to characterize populations of *Asimina triloba.* Measures of diversity are: *A*_T_ = total number of alleles, *A*_E_ = effective number of alleles, *H*_O_ = observed heterozygosity, *H*_E_ = expected heterozygosity, *F*_ST_ = Wright’s *F*_ST_ and *F*_IS_ = inbreeding coefficient.

| **Locus** | **Motif** | **Fragment size (bp)** | ***A*_T_** | ***A*_E_** | ***H*_O_** | ***H*_E_** | ***F*_ST_** | ***F*_IS_** |
| --- | --- | --- | --- | --- | --- | --- | --- | --- |
| Pp-G4 | AAT | 187 - 190 | 2 | 1.50 | 0.34 | 0.28 | 0.28 | -0.21 |
| Pp-G119 | AAT | 158 - 191 | 13 | 2.68 | 0.70 | 0.56 | 0.57 | -0.26 |
| Pp-G124 | AAT | 198 - 225 | 10 | 2.50 | 0.61 | 0.54 | 0.55 | -0.14 |
| Pp-C108 | ATG | 183 - 204 | 10 | 2.26 | 0.64 | 0.49 | 0.31 | -0.31 |
| Pp-G103 | AAT | 278 - 338 | 23 | 2.02 | 0.33 | 0.35 | 0.43 | 0.04 |
| Pp-G121 | AAT | 183 - 240 | 14 | 2.72 | 0.69 | 0.56 | 0.33 | -0.28 |
| Pp-B103 | GA | 262 - 360 | 44 | 3.68 | 0.68 | 0.62 | 0.35 | -0.11 |
| Pp-B108 | GA | 114 - 140 | 14 | 2.52 | 0.59 | 0.49 | 0.44 | -0.21 |
| Pp-B128 | GA | 137 - 193 | 25 | 2.33 | 0.36 | 0.43 | 0.55 | 0.16 |
| **Mean** | **-** | **-** | **17.2** | **2.47** | **0.55** | **0.48** | **0.42** | **-0.15** |
| ***SD*** | **-** | **-** | **12.2** | **0.59** | **0.16** | **0.11** | **0.11** | **0.16** |

**Table S3.**  Probability of identity (*PI*) values for 20 putative anthropogenic and 62 wild populations of *A. triloba*. N = number of samples and MLGs = number of multi-locus genotypes. * The number of MLGs differed significantly between anthropogenic and wild populations (*p* = 0.013; Figure S7).

| **Population** | **N** | **MLGs** | ***PI*** |
| --- | --- | --- | --- |
| **Anthropogenic** |  |  |  |
| FCP | 35 | 3 | 1.84E-04 |
| TUN | 21 | 7 | 1.32E-04 |
| OSF | 35 | 3 | 1.84E-04 |
| PET | 24 | 22 | 1.26E-04 |
| SEL | 5 | 2 | 2.63E-05 |
| MC3 | 12 | 1 | 6.32E-05 |
| MC2 | 18 | 10 | 9.49E-05 |
| MC1 | 16 | 7 | 8.43E-05 |
| POC | 48 | 6 | 2.53E-04 |
| FHM | 46 | 6 | 2.53E-04 |
| SCI | 24 | 1 | 1.26E-04 |
| LM3 | 14 | 3 | 7.38E-05 |
| LM2 | 17 | 14 | 1.26E-04 |
| LM1 | 24 | 1 | 8.96E-05 |
| SU1 | 33 | 11 | 1.74E-04 |
| CFP | 48 | 1 | 2.53E-04 |
| CON | 30 | 2 | 1.58E-04 |
| LCN | 48 | 4 | 2.53E-04 |
| CHA | 38 | 1 | 2.05E-04 |
| WEN | 10 | 1 | 5.27E-05 |
| **Mean** | **27.3** | **5.3** | **1.46E-04** |
| ***SD*** | **13.6** | **5.5** | **7.23E-05** |
|  |  |  |  |
| **Wild** |  |  |  |
| HAM | 47 | 2 | 2.48E-04 |
| SAN | 21 | 1 | 1.11E-04 |
| NAG | 29 | 3 | 1.58E-04 |
| HEM | 40 | 5 | 2.11E-04 |
| PCB | 48 | 14 | 7.20E-05 |
| CAD | 36 | 21 | 2.05E-04 |
| EUT | 33 | 32 | 5.00E-07 |
| BTH | 36 | 36 | 3.40E-07 |
| TAL | 24 | 3 | 8.90E-03 |
| COL | 48 | 18 | 7.50E-05 |
| SHE | 24 | 5 | 4.00E-03 |
| LCC | 24 | 23 | 2.20E-05 |
| TER | 33 | 27 | 3.20E-06 |
| LIL | 24 | 24 | 2.20E-05 |
| WBF | 43 | 22 | 5.10E-06 |
| WDP | 27 | 3 | 6.10E-03 |
| BCL | 48 | 12 | 6.00E-09 |
| LEE | 24 | 12 | 1.26E-04 |
| STO | 9 | 5 | 5.27E-05 |
| SAL | 30 | 14 | 4.10E-05 |
| MS1 | 24 | 4 | 1.26E-04 |
| MS2 | 23 | 1 | 1.21E-04 |
| CFR | 20 | 5 | 1.20E-05 |
| BCB | 25 | 8 | 1.32E-04 |
| CAT | 16 | 1 | 8.43E-05 |
| SHO | 24 | 16 | 1.26E-04 |
| KNX | 25 | 3 | 1.32E-04 |
| RL | 27 | 10 | 1.42E-04 |
| PER | 46 | 4 | 2.53E-04 |
| ER2 | 10 | 4 | 5.27E-05 |
| LHP | 20 | 4 | 1.05E-04 |
| LL1 | 25 | 1 | 1.32E-04 |
| LL2 | 25 | 7 | 1.32E-04 |
| BSF | 23 | 21 | 1.21E-05 |
| LL3 | 24 | 24 | 1.26E-04 |
| BLU | 25 | 9 | 9.50E-05 |
| MWR | 25 | 14 | 1.32E-04 |
| DRP | 23 | 13 | 1.32E-04 |
| OFR | 33 | 7 | 5.10E-06 |
| CKW | 22 | 13 | 1.74E-04 |
| HOL | 10 | 9 | 1.70E-04 |
| GCR | 48 | 5 | 5.27E-05 |
| KAN | 43 | 10 | 2.53E-04 |
| CCC | 26 | 12 | 2.27E-04 |
| CHR | 30 | 19 | 1.37E-04 |
| PWP | 48 | 27 | 6.20E-06 |
| RCA | 24 | 16 | 2.53E-04 |
| WSL | 11 | 9 | 4.27E-07 |
| MAN | 48 | 1 | 2.53E-04 |
| ROC | 25 | 1 | 1.32E-04 |
| MLK | 24 | 2 | 3.50E-02 |
| PIA | 14 | 1 | 7.38E-05 |
| PMQ | 59 | 4 | 3.16E-04 |
| LAW | 30 | 12 | 9.60E-03 |
| DCR | 15 | 1 | 7.90E-05 |
| BCP | 48 | 2 | 2.53E-04 |
| HPC | 25 | 15 | 1.12E-03 |
| TYG | 48 | 2 | 2.53E-04 |
| WES | 22 | 9 | 1.16E-04 |
| LOC | 38 | 3 | 2.00E-04 |
| SUS | 30 | 8 | 1.58E-04 |
| SFP | 25 | 7 | 1.32E-04 |
| **Mean** | **29.4** | **10.1** | **1.15E-03** |
| ***SD*** | **11.5** | **8.6** | **4.74E-03** |

**Table S4.**  Q-matrix generated by Structure for 20 putative anthropogenic (A) and 62 wild (W) populations of *A. triloba* from the run with highest Ln P(*D*) for *K* = 2 (-50942.4). Values in columns represent the posterior probabilities of assignment to one of two clusters. Clusters 1 and 2 correspond with the Eastern and Western clusters respectively.

| **Population** | **A/W** | **Cluster 1** | **Cluster 2** |
| --- | --- | --- | --- |
| TAL | W | 0.990 | 0.010 |
| COL | W | 0.988 | 0.012 |
| PCB | W | 0.983 | 0.017 |
| CAT | W | 0.981 | 0.019 |
| SHE | W | 0.980 | 0.020 |
| PIA | W | 0.979 | 0.021 |
| OSF | A | 0.978 | 0.022 |
| MAN | W | 0.972 | 0.028 |
| CFR | W | 0.945 | 0.055 |
| CFP | A | 0.939 | 0.061 |
| ER2 | W | 0.922 | 0.078 |
| LCC | W | 0.922 | 0.078 |
| LIL | W | 0.906 | 0.094 |
| SAL | W | 0.898 | 0.102 |
| POC | A | 0.885 | 0.115 |
| TER | W | 0.872 | 0.128 |
| KNX | W | 0.863 | 0.137 |
| PET | A | 0.752 | 0.248 |
| STO | W | 0.703 | 0.297 |
| BTH | W | 0.681 | 0.319 |
| SEL | A | 0.629 | 0.371 |
| WBF | W | 0.560 | 0.440 |
| PWP | W | 0.528 | 0.472 |
| NAG | W | 0.505 | 0.495 |
| LL2 | W | 0.401 | 0.599 |
| EUT | W | 0.356 | 0.644 |
| SHO | W | 0.312 | 0.688 |
| CCC | W | 0.276 | 0.724 |
| RCA | W | 0.210 | 0.790 |
| SUS | W | 0.123 | 0.877 |
| CKW | W | 0.054 | 0.946 |
| RL | W | 0.054 | 0.946 |
| MWR | W | 0.045 | 0.955 |
| SU1 | A | 0.045 | 0.955 |
| WSL | W | 0.037 | 0.963 |
| WDP | W | 0.033 | 0.967 |
| LEE | W | 0.029 | 0.971 |
| LOC | W | 0.028 | 0.972 |
| MC3 | A | 0.024 | 0.976 |
| HEM | W | 0.022 | 0.978 |
| KAN | W | 0.022 | 0.978 |
| CAD | W | 0.021 | 0.979 |
| MC1 | A | 0.015 | 0.985 |
| OFR | W | 0.014 | 0.986 |
| HPC | W | 0.013 | 0.987 |
| CHR | W | 0.013 | 0.987 |
| LL3 | W | 0.012 | 0.988 |
| BLU | W | 0.011 | 0.989 |
| DRP | W | 0.011 | 0.989 |
| BCL | W | 0.009 | 0.991 |
| HOL | W | 0.009 | 0.991 |
| LAW | W | 0.009 | 0.991 |
| LM1 | A | 0.009 | 0.991 |
| PER | W | 0.009 | 0.991 |
| FCP | A | 0.008 | 0.992 |
| HAM | W | 0.008 | 0.992 |
| WES | W | 0.008 | 0.992 |
| BCB | W | 0.008 | 0.992 |
| MC2 | A | 0.007 | 0.993 |
| GCR | W | 0.006 | 0.994 |
| BSF | W | 0.005 | 0.995 |
| SFP | W | 0.005 | 0.995 |
| FHM | A | 0.004 | 0.996 |
| CON | A | 0.004 | 0.996 |
| CHA | A | 0.004 | 0.996 |
| TUN | A | 0.003 | 0.997 |
| BCP | W | 0.003 | 0.997 |
| LHP | W | 0.003 | 0.997 |
| LL1 | W | 0.003 | 0.997 |
| LM2 | A | 0.003 | 0.997 |
| MS1 | W | 0.002 | 0.998 |
| MS2 | W | 0.002 | 0.998 |
| CAH | W | 0.002 | 0.998 |
| MLK | W | 0.002 | 0.998 |
| ROC | W | 0.002 | 0.998 |
| DCR | W | 0.002 | 0.998 |
| LM3 | A | 0.002 | 0.998 |
| SCI | A | 0.002 | 0.998 |
| TYG | W | 0.002 | 0.998 |
| WEN | A | 0.002 | 0.998 |
| LCN | A | 0.002 | 0.998 |
| SAN | W | 0.001 | 0.999 |
| PMQ | W | 0.001 | 0.999 |

**Table S5** Summary of fruit set on genotyped stems documented in 20 putative anthropogenic and 62 wild populations of *Asimina triloba*. Populations are arranged in order of ascending latitude. *N* = number of stems sampled and MLGs = number of multi-locus genotypes. In wild populations, higher fruit set is significantly correlated with the presence of more MLGs (*r* = 0.498; *p* = 3.77E-05), but the relationship was not significant for anthropogenic populations (*r* = 0.219; *p* = 0.354).

| **Population** | ***N*** | **MLGs** | **Fruits** | **Fruits/Stem** | **Fruits/MLG** |
| --- | --- | --- | --- | --- | --- |
| **Anthropogenic** |  |  |  |  |  |
| FCP | 35 | 3 | 0 | 0.00 | 0.00 |
| TUN | 25 | 7 | 11 | 0.44 | 1.57 |
| OSF | 36 | 3 | 0 | 0.00 | 0.00 |
| PET | 24 | 21 | 1 | 0.04 | 0.05 |
| SEL | 5 | 2 | 0 | 0.00 | 0.00 |
| MC3 | 12 | 1 | 0 | 0.00 | 0.00 |
| MC2 | 18 | 10 | 19 | 1.06 | 1.90 |
| MC1 | 16 | 7 | 2 | 0.13 | 0.29 |
| POC | 50 | 6 | 0 | 0.00 | 0.00 |
| FHM | 50 | 6 | 18 | 0.36 | 3.00 |
| SCI | 25 | 1 | 1 | 0.04 | 1.00 |
| LM3 | 14 | 3 | 0 | 0.00 | 0.00 |
| LM1 | 17 | 14 | 0 | 0.00 | 0.00 |
| LM2 | 24 | 1 | 0 | 0.00 | 0.00 |
| SU1 | 32 | 11 | 0 | 0.00 | 0.00 |
| CFP | 50 | 1 | 0 | 0.00 | 0.00 |
| CON | 30 | 2 | 0 | 0.00 | 0.00 |
| LCN | 48 | 4 | 1 | 0.02 | 0.25 |
| CHA | 40 | 1 | 0 | 0.00 | 0.00 |
| WEN | 10 | 1 | 0 | 0.00 | 0.00 |
| **Mean** | **28.1** | **5.3** | **2.7** | **0.10** | **0.40** |
| ***SD*** | **14.2** | **5.3** | **5.9** | **0.26** | **0.83** |
|  |  |  |  |  |  |
| **Wild** |  |  |  |  |  |
| HAM | 47 | 2 | 0 | 0.00 | 0.00 |
| SAN | 21 | 1 | 0 | 0.00 | 0.00 |
| NAG | 30 | 3 | 0 | 0.00 | 0.00 |
| HEM | 40 | 5 | 0 | 0.00 | 0.00 |
| PCB | 48 | 12 | 4 | 0.08 | 0.29 |
| CAD | 39 | 18 | 0 | 0.00 | 0.00 |
| EUT | 33 | 30 | 11 | 0.33 | 0.34 |
| BTH | 37 | 10 | 21 | 0.57 | 0.58 |
| TAL | 24 | 3 | 0 | 0.00 | 0.00 |
| COL | 48 | 12 | 18 | 0.38 | 1.00 |
| SHE | 24 | 5 | 0 | 0.00 | 0.00 |
| LCC | 24 | 18 | 42 | 1.75 | 1.83 |
| TER | 34 | 22 | 75 | 2.21 | 2.78 |
| LIL | 24 | 21 | 149 | 6.21 | 6.21 |
| WBF | 43 | 21 | 10 | 0.23 | 0.45 |
| WDP | 27 | 3 | 6 | 0.22 | 2.00 |
| BCL | 48 | 27 | 0 | 0.00 | 0.00 |
| LEE | 24 | 12 | 0 | 0.00 | 0.00 |
| STO | 10 | 5 | 0 | 0.00 | 0.00 |
| SAL | 30 | 14 | 7 | 0.23 | 0.50 |
| MS1 | 24 | 4 | 0 | 0.00 | 0.00 |
| MS2 | 23 | 1 | 0 | 0.00 | 0.00 |
| CFR | 20 | 5 | 10 | 0.50 | 2.00 |
| BCB | 25 | 8 | 0 | 0.00 | 0.00 |
| CAT | 16 | 1 | 0 | 0.00 | 0.00 |
| SHO | 24 | 16 | 14 | 0.58 | 0.88 |
| KNX | 25 | 3 | 1 | 0.04 | 0.33 |
| RL | 27 | 12 | 0 | 0.00 | 0.00 |
| PER | 48 | 5 | 3 | 0.06 | 0.75 |
| ER2 | 10 | 5 | 0 | 0.00 | 0.00 |
| LHP | 20 | 4 | 0 | 0.00 | 0.00 |
| LL1 | 25 | 1 | 0 | 0.00 | 0.00 |
| LL2 | 25 | 7 | 0 | 0.00 | 0.00 |
| BSF | 23 | 21 | 20 | 0.87 | 0.95 |
| LL3 | 24 | 24 | 2 | 0.08 | 0.08 |
| BLU | 25 | 9 | 0 | 0.00 | 0.00 |
| MWR | 25 | 14 | 0 | 0.00 | 0.00 |
| DRP | 23 | 13 | 4 | 0.17 | 0.31 |
| OFR | 33 | 6 | 0 | 0.00 | 0.00 |
| CKW | 24 | 13 | 21 | 0.88 | 1.62 |
| HOL | 10 | 8 | 0 | 0.00 | 0.00 |
| GCR | 48 | 6 | 3 | 0.06 | 0.60 |
| KAN | 43 | 10 | 0 | 0.00 | 0.00 |
| CCC | 26 | 13 | 0 | 0.00 | 0.00 |
| CHR | 30 | 18 | 18 | 0.60 | 0.95 |
| PWP | 48 | 32 | 12 | 0.25 | 0.44 |
| RCA | 25 | 16 | 0 | 0.00 | 0.00 |
| WSL | 11 | 9 | 0 | 0.00 | 0.00 |
| MAN | 48 | 1 | 0 | 0.00 | 0.00 |
| ROC | 25 | 1 | 0 | 0.00 | 0.00 |
| MLK | 24 | 2 | 0 | 0.00 | 0.00 |
| PIA | 14 | 1 | 0 | 0.00 | 0.00 |
| PMQ | 60 | 4 | 5 | 0.08 | 1.25 |
| LAW | 30 | 12 | 9 | 0.30 | 0.75 |
| DCR | 15 | 1 | 0 | 0.00 | 0.00 |
| BCP | 48 | 2 | 0 | 0.00 | 0.00 |
| HPC | 25 | 14 | 29 | 1.16 | 1.93 |
| TYG | 48 | 2 | 0 | 0.00 | 0.00 |
| WES | 22 | 9 | 0 | 0.00 | 0.00 |
| LOC | 38 | 3 | 0 | 0.00 | 0.00 |
| SUS | 30 | 8 | 4 | 0.13 | 0.50 |
| SFP | 25 | 7 | 0 | 0.00 | 0.00 |
| **Mean** | **29.6** | **9.6** | **8.0** | **0.29** | **0.47** |
| ***SD*** | **11.5** | **7.8** | **21.9** | **0.87** | **0.98** |

**Figure S1**. Comparison of the number of unique multi-locus genotypes found in anthropogenic and wild populations (*p =* 0.011). See Table S4.


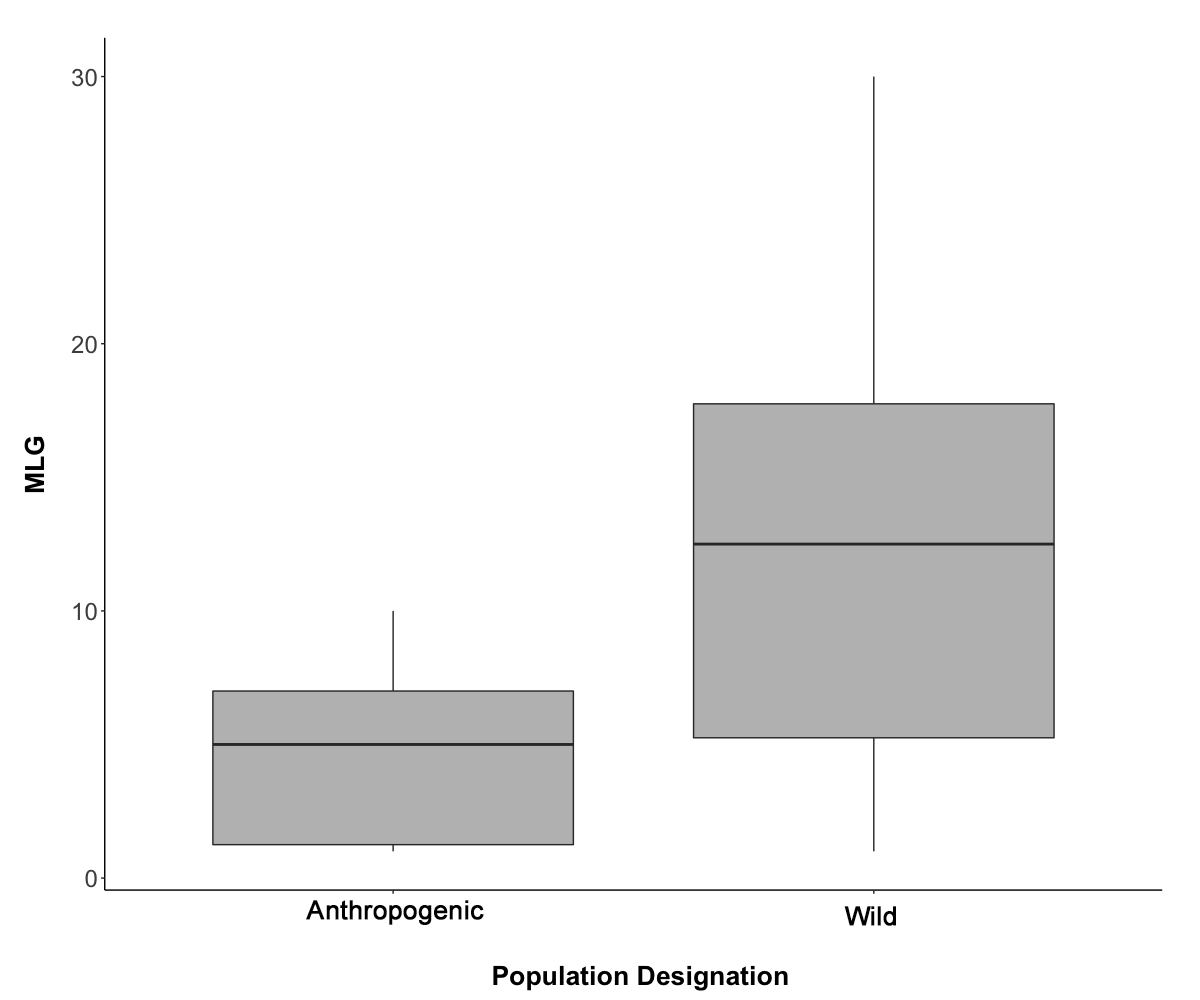


**Figure S2**. Graph showing the distribution of Δ*K* for *K* = 2 – 10 clusters generated in Structure Harvester for all 82 populations of *Asimina triloba*, yielding an optimal *K* = 2 clusters.

**
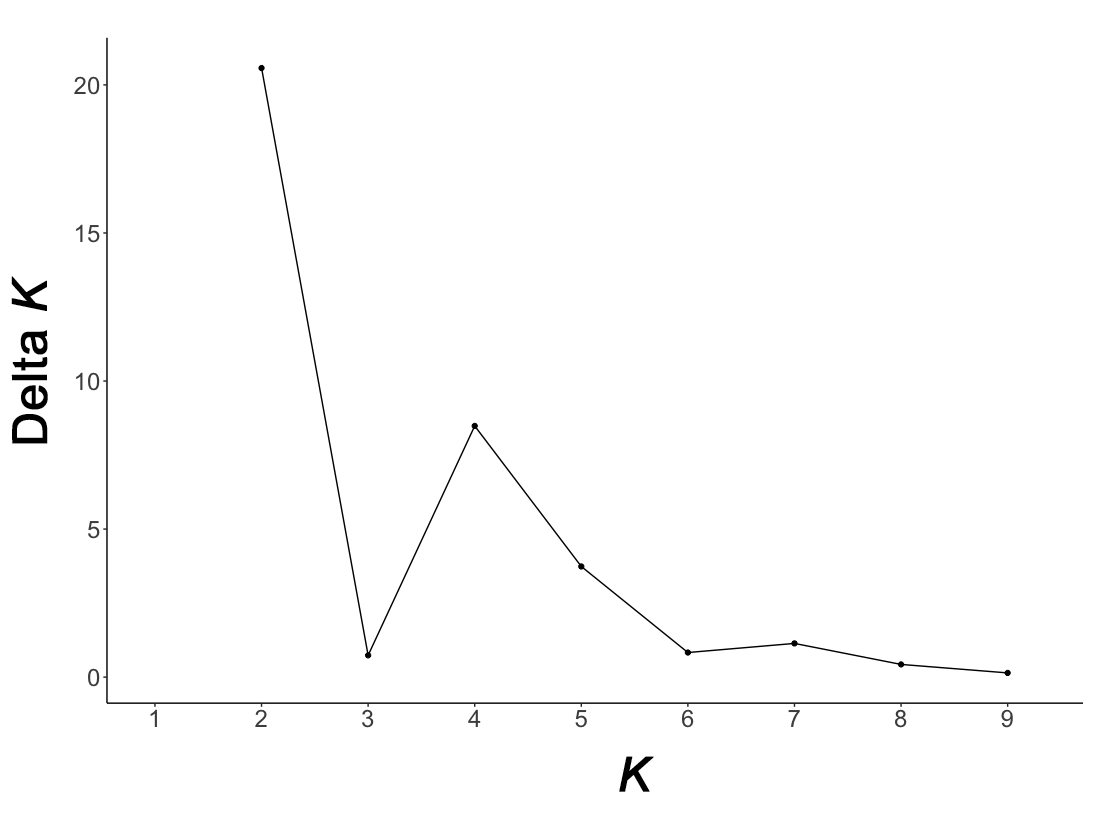
**

**Figure S3**. All sampled populations (*N* = 82) showing proportion of each population assigned to four clusters which is the second most likely *K* value yielded by Structure v 2.3.4. A = putative anthropogenic populations. Wild populations have no designation. The reported range of *A. triloba* by Little (1977) is outlined in blue.


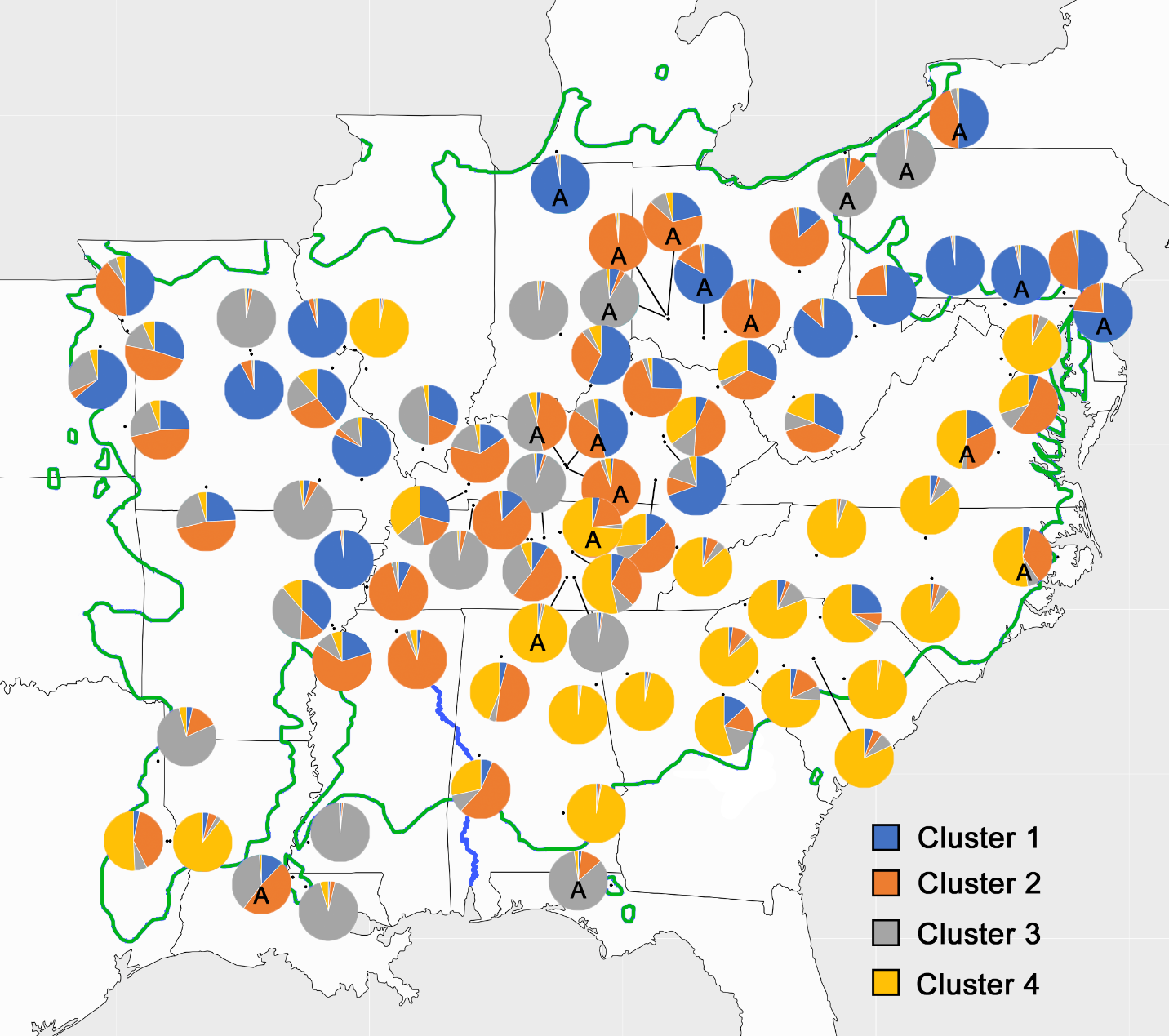


**Figure S4** Venn diagram showing rare alleles shared by all populations relative to the number of rare alleles shared by wild and anthropogenic populations.

**
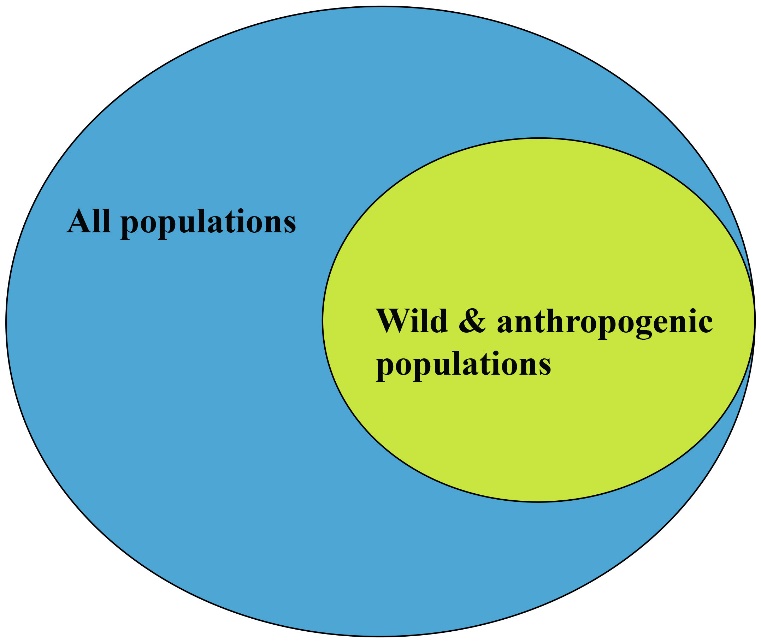
**
